# MicroRNA-134 Contributes to Glucose-Induced Endothelial Cell Dysfunction and This Effect Can Be Reversed by Far-Infrared Irradiation

**DOI:** 10.1371/journal.pone.0147067

**Published:** 2016-01-22

**Authors:** Hsei-Wei Wang, Shu-Han Su, Yen-Li Wang, Shih-Ting Chang, Ko-Hsun Liao, Hung-Hao Lo, Ya-Lin Chiu, Tsung-Han Hsieh, Tse-Shun Huang, Chin-Sheng Lin, Shu-Meng Cheng, Cheng-Chung Cheng

**Affiliations:** 1 Institute of Microbiology and Immunology, National Yang-Ming University, Taipei, Taiwan; 2 VGH-YM Genome Research Center, National Yang-Ming University, Taipei, Taiwan; 3 Department of Education and Research, Taipei City Hospital, Taipei, Taiwan; 4 Program in Molecular Medicine, National Yang-Ming University and Academia Sinica, Taipei, Taiwan; 5 Institute of Biomedical Informatics, National Yang-Ming University, Taipei, Taiwan; 6 Institute of Engineering in Medicine, University of California, San Diego, La Jolla, California, United States of America; 7 Division of Cardiology, Department of Internal Medicine, Tri-Service General Hospital, National Defense Medical Center, Taipei, Taiwan; Peking Union Medical College Hospital, CHINA

## Abstract

Diabetes mellitus (DM) is a metabolic disease that is increasing worldwide. Furthermore, it is associated with the deregulation of vascular-related functions, which can develop into major complications among DM patients. Endothelial colony forming cells (ECFCs) have the potential to bring about medical repairs because of their post-natal angiogenic activities; however, such activities are impaired by high glucose- (HG) and the DM-associated conditions. Far-infrared radiation (FIR) transfers energy as heat that is perceived by the thermoreceptors in human skin. Several studies have revealed that FIR improves vascular endothelial functioning and boost angiogenesis. FIR has been used as anti-inflammatory therapy and as a clinical treatment for peripheral circulation improvement. In addition to vascular repair, there is increasing evidence to show that FIR can be applied to a variety of diseases, including cardiovascular disorders, hypertension and arthritis. Yet mechanism of action of FIR and the biomarkers that indicate FIR effects remain unclear. MicroRNA-134 (miR-134-5p) was identified by small RNA sequencing as being increased in high glucose (HG) treated dfECFCs (HG-dfECFCs). Highly expressed miR-134 was also validated in dmECFCs by RT-qPCR and it is associated with impaired angiogenic activities of ECFCs. The functioning of ECFCs is improved by FIR treatment and this occurs via a reduction in the level of miR-134 and an increase in the NRIP1 transcript, a direct target of miR-134. Using a mouse ischemic hindlimb model, the recovery of impaired blood flow in the presence of HG-dfECFCs was improved by FIR pretreatment and this enhanced functionality was decreased when there was miR-134 overexpression in the FIR pretreated HG-dfECFCs. In conclusion, our results reveal that the deregulation of miR-134 is involved in angiogenic defects found in DM patients. FIR treatment improves the angiogenic activity of HG-dfECFCs and dmECFCs and FIR has potential as a treatment for DM. Detection of miR-134 expression in FIR-treated ECFCs should help us to explore further the effectiveness of FIR therapy.

## Introduction

Diabetes mellitus (DM) is one of a group of chronic metabolic disorders that are characterized by inappropriate hyperglycemia and it has been estimated that the disease will affect 7.8% of the world adult population by 2030 [1]. The predominant form of diabetes is type 2 (T2D), which is accounts for nearly to 90% of cases, with the other 10% consisting of type 1 diabetes and gestational diabetes (GDM). In addition to hyperglycemia, the presence of high glucose impaired blood vessels and aberrant angiogenesis that contribute to many of the clinical manifestations of diabetes and are important caused of mortality among DM patients [2]. Thus it is important to identify protective approaches and therapeutic strategies that minimize the complications of DM.

Endothelial colony forming cells (ECFCs, also called late outgrowth endothelial cells, OECs) are circulating endothelial cells that express endothelial lineage surface antigens such as platelet/endothelial cell adhesion molecule 1 (PECAM1), cadherin 5 type 2 (vascular endothelium) (CDH5, also called VE-cadherin), kinase inset domain receptor (KDR, also called VEGFR2) and hematopoietic stem cell marker (CD34), but lack hematopoietic lineage markers (CD14 and CD45) [3, 4]. ECFCs are potential tissue engineering materials because they show significant proliferation and *de novo* tubulogenic ability [5]. ECFCs are capable of being incorporated into the resident vasculature directly and consequently help recovery of damaged vascular regions in *in vivo* ischemia models [3, 6–8]. ECFCs after high glucose treatment or isolated from GDM pregnancies show a slower cell proliferation rate, impaired cell migration ability and poorer tube formation ability [9, 10]. An investigation into the molecular mechanisms at work in ECFCs from a diabetic environment or when grown in high glucose conditions should aid our understanding of how to bring about therapeutic improvements in angiogenesis.

MicroRNAs (miRNAs) are small non-coding RNA molecules that are 21~23 nucleotides in length and are crucial for posttranscriptional gene regulation [11]. miRNAs mediate a wide range of cellular processes by inhibiting their targets through either translational repression or mRNA degradation. Dysregulation of miRNAs has been observed to be associated with a range of human pathologies including cancer, neurodegeneration and vascular diseases [12–15]. miRNAs are able to target pro-angiogenic or anti-angiogenic factors that play critical roles in controlling angiogenesis. In DM, several miRNAs have been identified as being involved in the regulation of pancreatic beta-cells; they do this by modulating cell growth, by controlling insulin biosynthesis and secretion, or by targeting tissues on which insulin acts, including liver, muscles and adipocytes [16]. Microvascular and macrovascular complications, which are characteristics of blood vessel damage and ischemic events, are the major complications in diabetic patients. miRNAs are dysregulated during the process of tissue hypoxia and manipulating these miRNAs improves postischemic revascularization. In mouse myocardial infarction model, intramyocardially injection of miR-210 precursor improves vascularization [17]. miR-100 is downregulated after femoral artery occlusion and inhibition of miR-100 restores perfusion in hindlimb ischemic region *in vivo* [18]. In this study, we evaluate the effect of miRNA in ECFC mediated restoration of blood flow perfusion using mouse hindlimb ischemia model. However, the regulatory mechanisms of miRNAs within DM and the consequent effects to ECFCs remain largely unclear.

Far infrared radiation (FIR) is an invisible form of electromagnetic energy that has a wavelength that is longer than visible light. There are three main techniques can be used for FIR radiation delivery, namely FIR saunas, FIR ray devices and FIR emitting ceramics and fabrics [19]. FIR is a promising treatment strategy for a number of medical conditions, such as improvement of sleep [20], inhibition of chronic pain [21]. FIR has also been shown to improve endothelial function, peripheral arterial disease and coronary artery-related ischemia [22–26]. In addition, FIR treatment promotes blood flow recovery after ischemia surgery in streptozotocin-induced diabetic mice. However, whether FIR improves the functional properties of ECFC among DM patients by allowing the repair of ischemia region and whether miRNAs contribute to FIR mediated restoration of blood flow remain largely unknown.

In this study, we investigated the role of miRNAs in impairing the functional abilities of ECFC under DM and high glucose (HG) condition and the decreased angiogenic activities can be reversed by FIR. Our results provide insights into the miRNA regulation that underlies the FIR treatment of HG-dfECFCs, and also reveals a new clue that how the FIR effect promotes HG-dfECFC activity during recovery from ischemic situation.

## Materials and Methods

### Ethics statement

This study was approved by the Institutional Ethics Committee/Institutional Review Board of Taipei City Hospital Heping Fuyou Branch (IRB number: TCHIRB-1030507) and Tri-Service General Hospital (IRB number: 1-103-05-061). Patients diagnosed with Type II DM are included in this study. All participants provide their written informed consent to participate in this study. The protocols of this study are consistent with the ethical guidelines of the 2008 Helsinki Declaration. Animal experimental procedures were approved by the Institutional Animal Care and Use Committee (IACUC) of National Yang-Ming University and are in compliance with the ARRIVE guidelines.

### Isolation of peripheral blood ECFCs from DM patients and disease-free donors

ECFCs were collected from the peripheral blood samples of disease-free (DF) subjects or DM patients and were isolated by density centrifugation using Histopaque-1077 (1.077 g/ml, Sigma, St. Louis, MO, USA). Isolated mononuclear cells (MNCs) were cultured in fibronectin-coated plates with Endothelial Growth Medium-2 (EGM-2; Lonza Ltd) containing complete supplement (Hydrocortisone, IGF-1, hEGF, hVEGF, hFGF2, ascorbic acid, GA-1000, heparin and 2% FBS). After 4–5 days of cultivation, the unattached cells were removed and the culture medium was refreshed. At 2–3 weeks, the attached ECFCs had a cobblestone-shaped appearance with a monolayer growth pattern. These ECFCs expressed both endothelial and hematopoietic stem cell surface markers as described earlier [3, 4, 27]. The ECFCs used in our experiments were from passage 2 to passage 6 in order to avoid any aging or senescence issues. In order to imitate the physiological DM condition, ECFCs were treated with 0.5M D-glucose stock solution in EGM-2 to the final concentration at 25 mM to form the high glucose (HG) group, which mimics hyperglycemia. Similarly, ECFCs were cultured in EBM-2 containing 2% FBS to form the low growth factor (LGF) group. In addition, ECFCs were cultured in LGF combined with 25 mM D-glucose (HG/LGF) to mimic advanced DM when hyperglycemia is accompanied by ischemia-induced tissue starvation. Finally, ECFCs were treated with D-mannitol to form the osmotic control group. The concentration of 25 mM D-glucose used for treatment is equivalent to the 450 mg/dl in human blood and this mimics that situation in type II DM patients [28].

### Isolation of human umbilical vein endothelial cells (HUVECs)

The umbilical cord vein perfused and digested with collagenase for 30 minutes at 37°C. DMEM with 10% FBS was used to drain out the endothelial cells from the lumen of the vein and these were then centrifuged. The cells were cultured in fibronectin-coated flasks with M199 medium containing 20% FBS, 5 μg/ml heparin, and 100 μg/ml ECGs. After 6 hours, the HUVECs had attached and the medium was replaced by EGM-2 (Lonza Ltd).

### Quantitative RT-PCR

RNA from cell lysates and plasma were extracted using Trizol® and Trizol® LS reagent (Life Technologies) respectively, according to the manufacturer's instructions [29]. For detection of miRNA expression, the expression levels of specific miRNAs were detected using the stem-loop strategy described in a previous study [30]. The specific primers for each miRNAs are listed in S1 Table. In order to measure gene expression, cDNA was synthesized using a RevertAid First Strand cDNA Synthesis Kit (Thermo Scientific). All the specific products of the miRNAs and genes were detected using Maxima™ SYBR Green qPCR Master Mix (Thermo Scientific) on a StepOne™ sequence detector (Applied Biosystems, USA). The miRNA expression levels from the ECFCs and plasma were normalized against U48 and miR-16, respectively [27, 31]. The mRNA expression levels were normalized against average GAPDH level.

### Gene and miRNA overexpression/knockdown

In order to overexpress various miRNAs, the precursor sequences of the miRNAs were cloned into lentiviral expression plasmids using the primer pairs listed in S2 Table. For NRIP1 knockdown, the shRNA was obtained from the National RNAi Core Facility in Academia Sinica, Taiwan. The plasmids for miRNA overexpression and NRIP1 knockdown were packaged into lentiviral particles using 293T cells and the protocol is briefly described below. A total of 2 μg pMDG, 2 μg pCMVDR8.91 and 3 μg Lenti-miRNA or shRNA plasmid were cotransfected into human embryonic kidneys (HEK)-293T cells using 14 μl Turbofect (Thermo Scientific™ Fermentas™, USA) regent. After 48 hours, the supernatant was collected and preserved at -80°C until further use. ECFCs seeded in 6-well plate with 80% confluency were used for supernatant infection and media were replaced with EGM2 containing complete supplements after 6 hours. The overexpression or knockdown efficiencies were evaluated 48 hrs post infection using qPCR.

To knock down miR-134 in ECFCs, commercial synthetic antagomir with chemical modification (2'-OMe-RNA backbone, first two and last four bases phosphorothioated, 3’-cholesterol tail) (RiboBio Co., Guangzhou, China) were added into culture medium at a final concentration of 50 nM at 70~80% cell confluence. The expression level of miR-134 was measured by qPCR after 48 hours.

### Transwell cell migration assay, tube formation assay, cell proliferation assay and cell cycle analysis

For the Transwell migration assay, 600 μl EGM2 medium with 10% FBS was added to the lower chamber, while 5 x 10^4^ ECFCs in 100 ul EGM2 medium was seeded into the upper 8 μm Polycarbonate Permeable Transwell chamber; the unit was then incubated at 37°C. After 6 hours, the supernatant was removed from the upper chamber and the 8μm membranes fixed with 4% paraformaldehyde for 15 minutes at room temperature. Hoechst 33342 reagent (Sigma-Aldrich) was added for 30 minutes to carry out nuclear staining. Migrated cells were counted under fluorescent microscopy across five representative fields.

For the *in vitro* tube formation assay, 50 μl thawed Basement Membrane Extract (Trevigen Inc.) was coated on 96-well plates at 37°C for 1 hour to form a reconstituted basement membrane. Next 7.5 x 10^3^ cells in 100 μl EGM-2 were seeded on the Matrigel and incubated at 37°C for 3–6 hours. Tube structures were then inspected under an inverted light microscope (10 X) and five representative fields in each group were captured in order to calculate total tube length. The total tube lengths were analyzed by ImageJ and are presented as relative tube length after normalization against control group.

For the MTT cell proliferation assay, 1 x 10^4^ cells in EGM-2 per well were cultured in fibronectin coated 24 well plates. The cell number was evaluated from day 0 to day 6. 450 μl MTT reagent (Invitrogen, USA) was added and incubated at 37°C. After 2.5 hours, MTT reagent was removed, 500 μl of 0.1% sodium dodecyl sulfate (SDS)-isopropanol solution was added and the formazan was separated out. A total of 200 μl solution from each well was transferred to 96-well plate and was determined using ELISA reader at 570 nm and 650 nm.

For cell cycle analysis, 1 x 10^6^ cells were fixed in cold 70% alcohol at -20°C overnight. Next 0.1% TritonX-100 containing 0.2 mg/ml RNase was used to permeabilize the cells for 1.5 hours at 37°C. Cells were then washed using PBS and stained with propidium iodide (PI) for 30 minutes. The cell cycle analysis was then carried out on a FACACanto (BD Pharmingen) and calculated by FlowJo.

Each experiment was examined with at least three unique patient lines and two replicates per lines in each functional assay.

### FACS analysis

ECFCs were suspended in PBS and 1 x 10^6^ cells was used for antibodies staining for 30 minutes at 4°C. The antibodies used were as follow and according to the manufacturer’s instructions: FITC-conjugated anti-CD34 (BD Pharmingen), FITC-conjugated anti-CD45 (Biolegend), PE-conjugated anti-VEGFR2 (R&D system), FITC-conjugated anti-CD31 (Miltenyi Biotech) and FITC-conjugated anti-VE-cadherin (AbD serotec). The cells were filtered using a 40 μm cell strainer and transfer to a Falcon tube for FACSCanto (BD Pharmingen) analysis and the cell surface markers as expressed percentages were analyzed by FlowJo.

### Far infrared radiation treatment

The cells were exposed to radiation from a WS^TM^ TY301 FIR emitter (Far IR Medical Technology, Taipei, Taiwan). The wavelength of the light generated from the electrified ceramic plates ranged from 5 to 12 μm with a peak at 8.2 μm. The radiator was placed 30 cm above the bottom of the culture plates for the indicated times. The functional assays were preformed 24 hours after FIR treatment.

### Small RNA sequencing (smRNA-seq) and data analysis

Total RNA was collected from one disease free ECFCs (dfECFC) and one high glucose treated dfECFCs (HG-dfECFCs). Small RNA fractions were sequenced using the Illumina GAIIx system (Illumina, San Diego, CA USA) following the manufacturer’s standard procedure. Sequencing data were analyzed by a published in-house bioinformatics pipeline [32]. The pipeline was conducted to process mRNA contamination, to identify known miRNA quantification, and to carry out format conversion. The RPM (reads per mapped reads) value for each miRNA was calculated in order to score its expression abundance. SVMicrO (compgenomics.utsa.edu/svmicro.html) [33] and miRTar (mirtar.mbc.nctu.edu.tw/) were [34] were used for miRNA downstream targets prediction.

### Reporter assays

In order to create the luciferase reporter plasmids by cloning, the putative miRNA-binding site was cloned into the XbaI site of the pGL3-Basic plasmid (Promega), while a mutated binding site was generated by OuickChange™ Site-Directed Mutagenesis Kit (Stratagene). The primers used are listed in S2 Table. For detection of luciferase intensities, miRNA and reporter plasmids were co-transfected into 293T cells using Turbofect reagent (Thermo Scientific™). Cell lysates were collected after 48 hours and their luciferase activities measured using a Dual-Luciferase Reporter Assay Kit (Promega). The luciferase activities were calculated based on the ratio of firefly luciferase to renilla luciferase activity and further normalized against the pGL3 vector control group.

### The mouse ischemic hindlimb model and ECFCs transplantation

Nude mice at 8 to 10 weeks of age received right femoral artery excision surgery to induce unilateral hindlimb ischemia as previously described [35]. The mice were anesthetized by an intraperitoneal injection of Tribromoethanol (Avertin®) and the right femoral artery was excised. Mice were arbitrarily arranged into four groups for different treatments: EGM2 medium, HG-dfECFCs, FIR treated HG-dfECFCs and FIR treated HG-dfECFCs with miR-134 overexpression. ECFCs were pre-stained with PKH26 (Sigma-Aldrich) for 30 minutes and transplanted at 3 days after surgery. Next 2.5 × 10^5^ ECFCs in 60 μl medium were injected intramuscularly at six different sites on the ischemic limb distal to the arterial occlusion site [36]. Blood perfusion was determined by Laser Doppler Perfusion Imager system (LDPI, Moor Instruments Limited, Devon, UK) before surgery, immediately after surgery and weekly following surgery. Blood flow was analyzed by moor LDI software version 5.3 and the results are indicated as the ratio of perfusion in the ischemic (right) versus non-ischemic (left) limb in each mouse. Cell counting and capillary density were analyzed using ImageJ by determining the PKH26/CD31 double positive cells and CD31 positive region, respectively.

### Immunofluorescence staining

Frozen section slides were treated with 200 ul of 3% H_2_O_2_ for 15 minutes at room temperature and were blocked with 5% BSA in 0.2% PBST for 1 hr. Then, slides were washed with PBS for 5 minutes for 3 times with shacking. A α-mouse CD31 antibody (Santa Cruz) in 0.2% BSA-PBST was used for detection of capillary.

### Statistical analyses

The results were statistically analyzed using the software SPSS v20. For experiments consist of two groups of samples, Mann-Whitney U test was used to evaluate the statistical significance. One-way ANOVA followed by Tukey’s post-hoc test was applied to determine the significance among each group of all double manipulation assays and other experiments with more than two treatment groups. A *p* value that is lower than 0.05 was considered statistically significant.

## Results

### High glucose impairs the functioning of ECFCs

**Fig 1:**
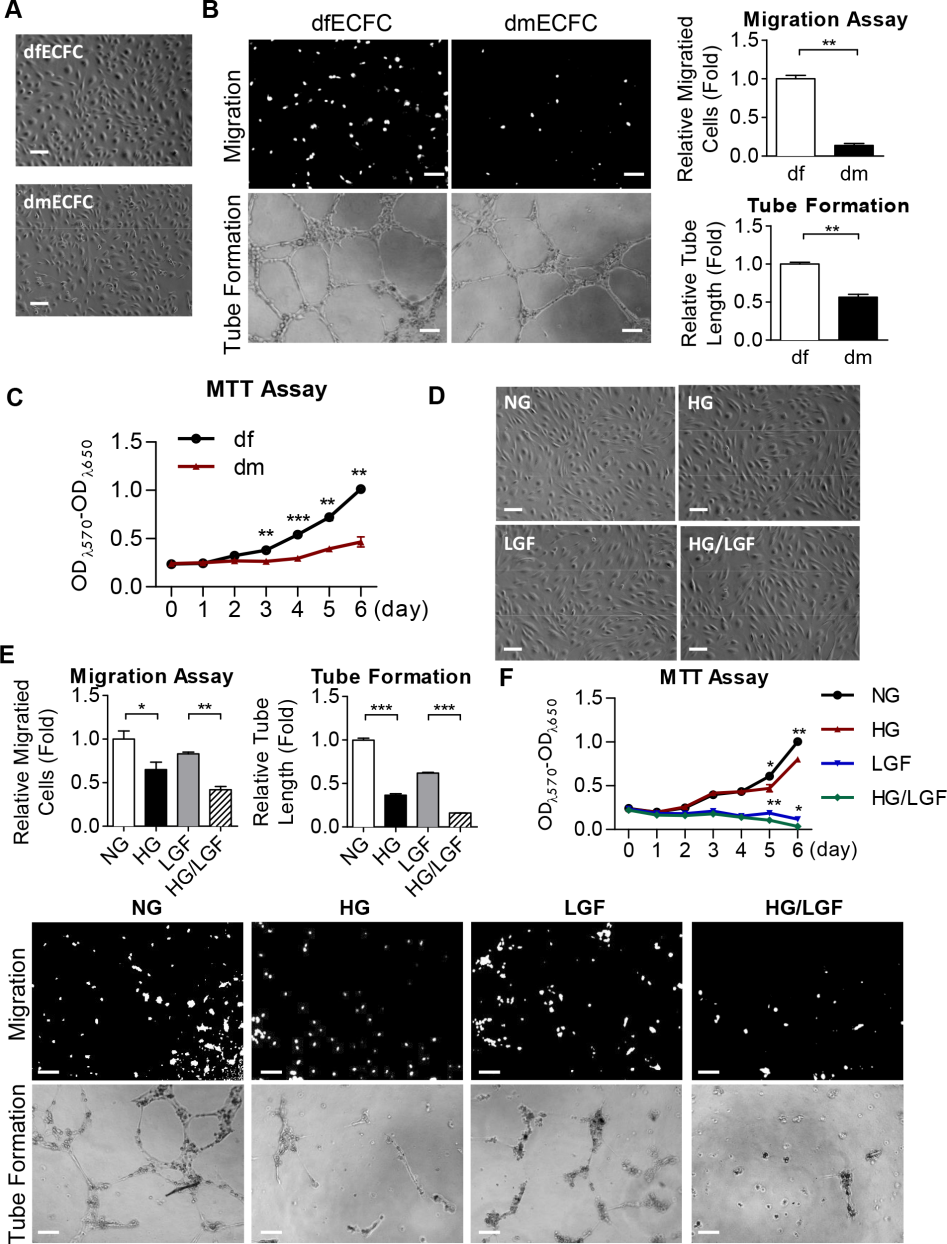
Reduced angiogenic activities in dmECFCs and high glucose treated dfECFCs. **(A)** The cell morphology of ECFCs from disease-free (df) and diabetes mellitus (dm) individuals Scale bar: 50 μm. **(B)** Representative images (left) and quantitative data (right) for the Transwell cellular migration assay (upper) and microvascular formation assay (lower) using dfECFCs and dmECFCs. n = 3 independent experiments. ** *p* < 0.01 by Mann-Whitney U test. Scale bar: 50 μm **(C)** Cell proliferation rates of dfECFCs and dmECFCs measured using the MTT assay. ** *p* < 0.01, *** *p* < 0.001 by Mann-Whitney U test. n = 3 independent experiments. **(D)** Cell morphology of dfECFCs treated with normal culture (NG), high glucose (HG), low growth factor (LGF) and HG/LGF. Scale bar: 50 μm **(E)** Representative images (lower) and quantitative data (upper) of Transwell migration assay and microvascular formation assay using dfECFCs under NG, HG, LGF, and HG/LGF conditions. n = 3 independent experiments. * *p* < 0.05, ** *p* < 0.01, *** *p* < 0.001 by one-way ANOVA followed Tukey’s post-hoc test. **(F)** Cell proliferation rate of dfECFCs under NG, HG, LGF, HG/LGF treatment measured using the MTT assay. n = 3 independent experiments. * *p* < 0.05, ** *p* < 0.01, by by one-way ANOVA followed by Tukey’s post-hoc test.

To investigate the functional activities of ECFCs in DM patients, we first isolated ECFCs from the peripheral blood of DM patients (dmECFCs) or disease-free subjects (dfECFCs). These two types of ECFCs both had a cobblestone-like morphology that was similar to mature endothelial cells [3, 4] (Fig 1A). Using flow cytometry analysis, both dm- and df-ECFCs expressed the precursor marker CD34 and endothelial markers, PECAM1, VE-cadherin and VEGFR2, but lacked hematopoietic lineage marker CD45 (S1A Fig). To assess their functioning, we compared the Transwell migration and tube formation ability of dmECFCs to df-ECFCs and found that dmECFCs showed 80% and 30% reduction in cell motility and the formation of microvasculature structure, respectively (Fig 1B). dmECFCs also proliferated 30% slower than that of dfECFCs on day 3 and this difference had further increased to 55% on day 6, as measured by MTT assay (Fig 1C).

In order to explore whether the impaired functioning of ECFCs was caused by high glucose, the isolated dfECFCs were treated for 72 hours with high glucose (HG) alone to mimic the hyperglycemia found in DM patients, or HG combined with low growth factor (LGF) conditions (HG/LGF) to mimic the poorest progression of DM patients. dfECFCs were also treated with D-mannitol (NG) and LGF used as control group for the HG and HG/LGF group, respectively. [28]. The morphologies (Fig 1D) and the expressed surface markers (S1B Fig) of all 4 groups of treated cells remained similar. Comparing to the NG and LGF groups, the HG and HG/LGF groups showed 35% and 40% decreases in cell migration, and also 63% and 74% reductions in tube formation ability, respectively (Fig 1E). Using the MTT assay, the HG and HG/LGF groups had a slower cell proliferation rate on day 5, with reductions of 23% and 45%, respectively (Fig 1F). However, a similar cell cycle patterns for these groups was found using flow cytometry, which indicates that the decreased cell proliferation was not due to cell cycle arrest (S2A Fig). Similar decreases in cell motility and tube formation caused by HG treatment were found for HUVECs (S2B Fig). These findings suggested that HG impairs the angiogenic activity of ECFCs.

### Small RNA sequencing analysis identified a set of miRNAs that were increased in HG-dfECFCs

**Fig 2:**
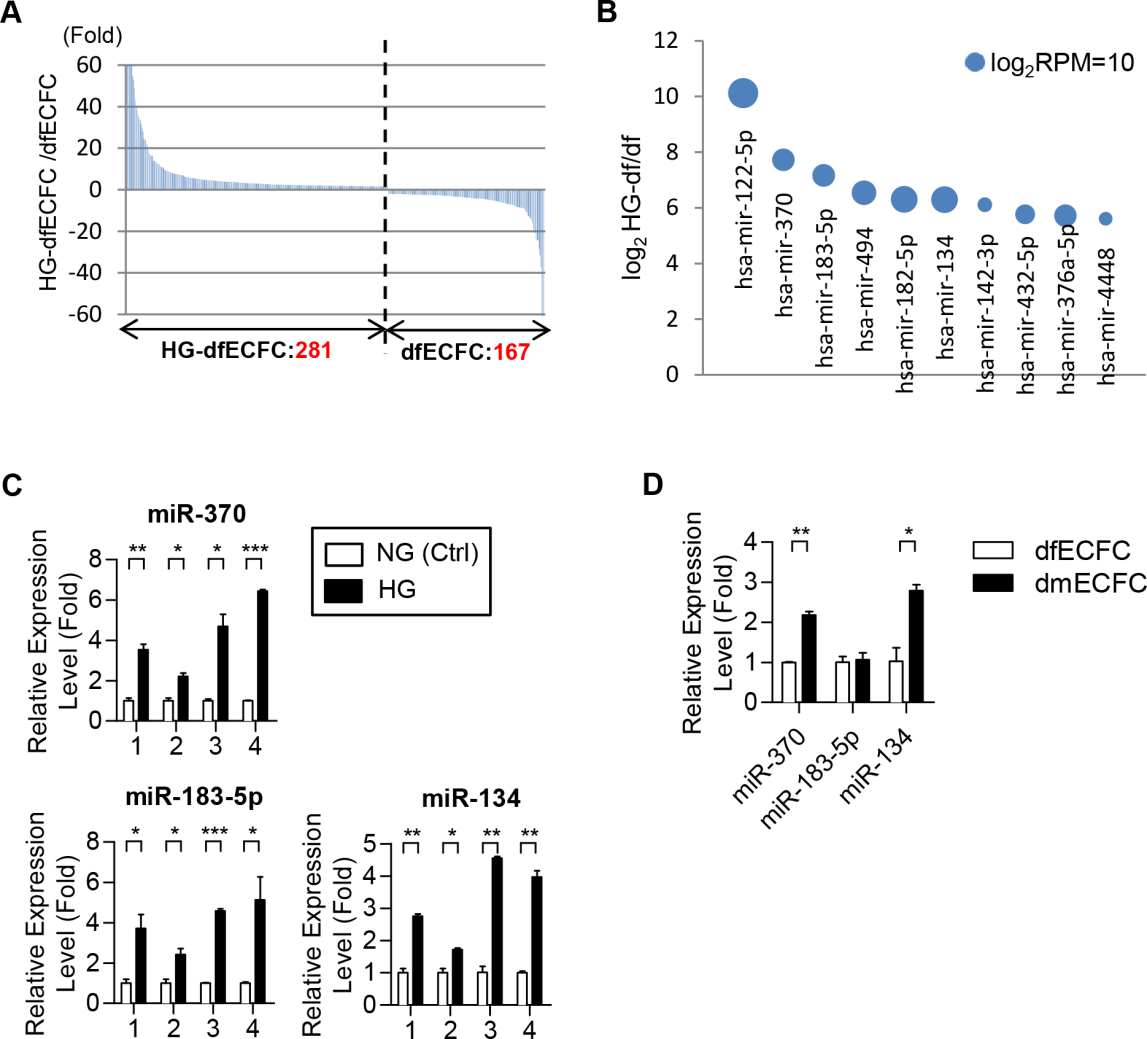
Small RNA sequencing and RT-qPCR reveal high level of expression of miR-134 in HG-dfECFCs and dmECFCs. **(A)** Differentially expressed miRNAs between HG-dfECFCs and dfECFCs. Numbers of dominant miRNAs in each ECFC type (fold change > 2) are shown. Y-axis was shown the fold ratio of RPM of HG-dfECFCs against dfECFCs. **(B)** The top ten HG-enriched miRNAs are shown. Y-axis was indicated the log_2_ ratio of HG-dfECFC against dfECFCs. The diameters of the circles indicate the log_2_RPM of the HG-dfECFCs. **(C)** The putative HG-induced miRNAs were validated using dfECFCs treated with NG and HG by RT-qPCR. * *p* < 0.05, ** *p* < 0.01, *** *p* < 0.001 by one-way ANOVA followed by Tukey’s post-hoc test. **(D)** The HG-induced miRNAs were also analyzed in dmECFCs and dfECFCs by RT-qPCR. * *p* < 0.05, ** *p* < 0.01 by one-way ANOVA followed by Tukey’s post-hoc test.

In order to explore the mechanisms that HG reduces the angiogenic related activities of ECFCs, we focused on the differences in miRNA expression patterns between HG-dfECFCs and dfECFCs using small RNA sequencing; our results were analyzed using our in-house bioinformatics pipeline [32]. We hypothesized that a set of miRNAs induced by HG hampered the functioning of dfECFCs. In summary, 448 known miRNAs were found to be differentially expressed between HG-dfECFCs and dfECFCs, with 281 and 167 miRNAs being more abundant in HG-dfECFCs and dfECFCs, respectively (> 2 fold; Fig 2A). The top ten HG-enriched miRNAs are shown as a bubble chart (Fig 2B and Table 1). We validated these miRNA expression levels using four DF subjects’ ECFCs incubated under NG and HG condition and found that miR-370, miR-183-5p and miR-134 were increased under the HG condition compared to the NG treatment condition (Fig 2C), whereas the other seven miRNAs showed no statistically significant change (S3 Fig). Only miR-370 and miR-134 were used for further experiments because these two miRNAs were found to be more abundant in dmECFCs compared to dfECFCs (Fig 2D).

**Table 1:** Top 10 enriched miRNAs in HG-dfECFCs.

	RPM		Fold
Name	dfECFC	HG-dfECFC	HG-df / df
hsa-mir-122-5p	84.143196	93717.86	1113.790116
hsa-mir-370	23.864511	5048.3394	211.5417073
hsa-mir-183-5p	38.978706	5603.7925	143.7654831
hsa-mir-494	119.32256	11111.615	93.12249922
hsa-mir-182-5p	320.84512	25406.03	79.18471691
hsa-mir-134	330.12573	25924.89	78.53035266
hsa-mir-142-3p	3.8044875	262.93384	69.11150056
hsa-mir-432-5p	34.240387	1866.4335	54.50970808
hsa-mir-376a-5p	95.88805	5047.9097	52.64378304
hsa-mir-4448	3.7865787	184.72424	48.78394314

The reads of each miRNAs was normalized with total reads and indicated as reads per million (RPM). Fold is the reads of HG-dfECFC against dfECFC.

### miR-134 regulates ECFC motility and microvasculature formation ability

**Fig 3:**
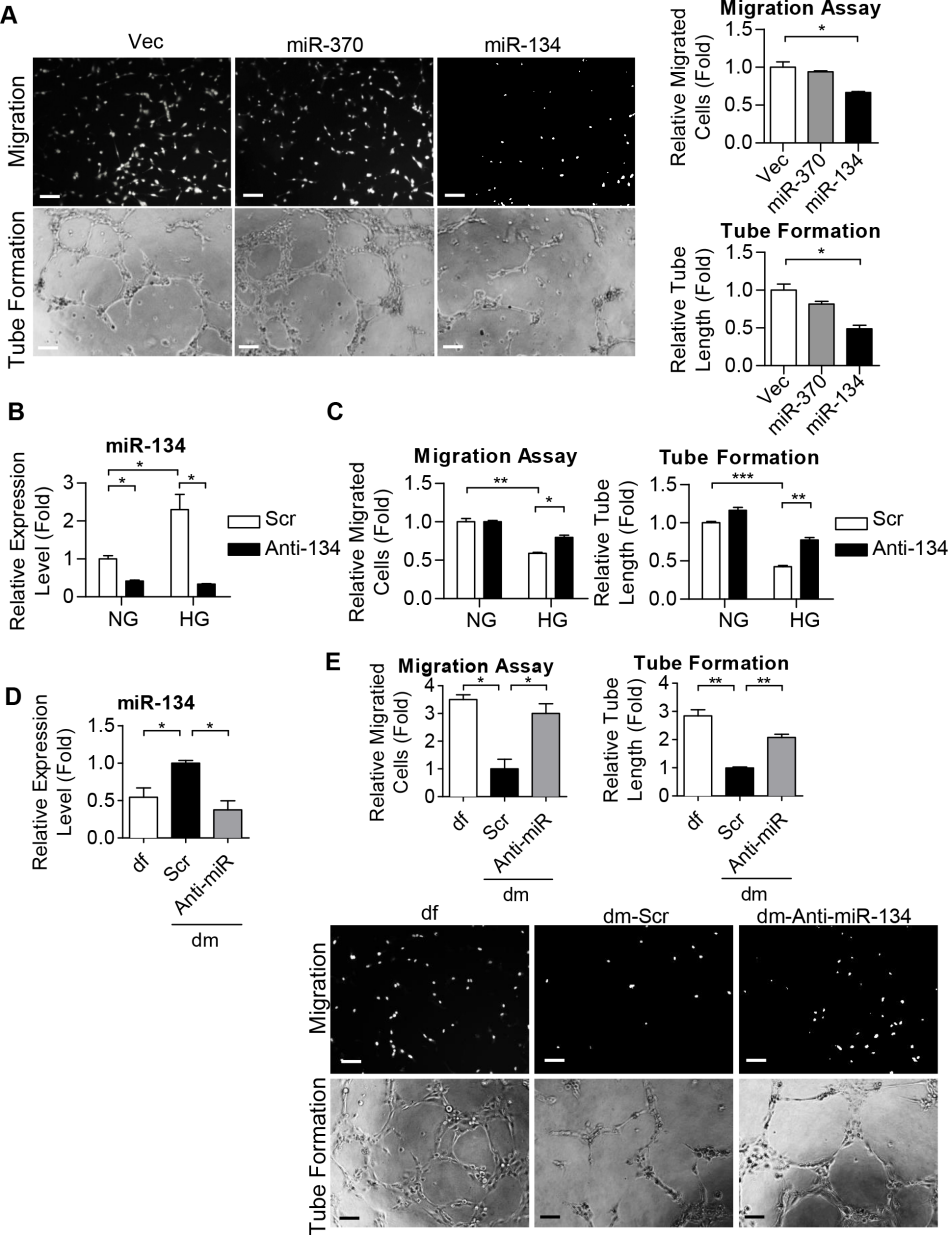
miR-134 regulates ECFC motility and microvasculature formation ability. **(A)** Representative images (left) and quantitative data (right) from the Transwell migration assays (upper) and tube formation assays (lower) of dfECFCs with miR-370 and miR-134 overexpression. n = 3 independent experiments. * *p* < 0.05 by one-way ANOVA followed by Tukey’s post-hoc test. Scale bar: 50 μm **(B)** The expression levels of miR-134 under osmotic culture condition (NG) or under high glucose condition (HG) treated with scramble control and miR-134 antagomir (Anti-134) in dfECFCs as quantified by RT-qPCR. * *p* < 0.05 by one-way ANOVA followed by Tukey’s post-hoc test n = 3 independent experiments. **(C)** Cellular migration assays (left) and tube formation assays (right) of NG-dfECFCs and HG-dfECFCs after scramble control and miR-134 antagomir (Anti-134) treatment. n = 3 independent experiments. * *p* < 0.05, ** *p* < 0.01, *** *p* < 0.001 by one-way ANOVA followed by Tukey’s post-hoc test. **(D)** Expression levels of miR-134 in dfECFCs or antagomir treated dmECFCs using RT-qPCR. n = 3 independent experiments. * *p* < 0.05 by one-way ANOVA followed by Tukey’s post-hoc test. **(E)** Representative images (lower) and quantitative data (upper) from the Transwell migration assays and tube formation assays of dfECFCs and antagomir treated dmECFCs. n = 3 independent experiments. * *p* < 0.05, ** *p* < 0.01 by one-way ANOVA followed by Tukey’s post-hoc test. Scale bar: 50 μm. Vec, plasmid control; Scr, scramble; Anti-134, miR-134 antagomir.

We next investigated the angiogenic functioning of miR-370 and miR-134 in dfECFCs. After overexpression of these two miRNAs in dfECFCs, miR-370 and miR-134 were 11-fold and 3.6-fold increased respectively (S4A Fig). Cell motility and microvasculature formation were then determined and it was found that miR-134 reduced cell migration by 34% and tube formation ability by 51%, while overexpression of miR-370 in dfECFCs resulted in no significant change (Fig 3A). Both miR-370 and miR-134 had no effect on cell proliferation or on cell cycle arrest (S4B and S4C Fig). We also measured the effect of miR-370 and miR-134 overexpression on cell motility and microvasculature formation of HUVECs (S4D Fig). The findings were similar to that of ECFCs with only miR-134 overexpression leading to a decrease in cell migration and tube formation (S4E Fig).

Next we used an oligonucleotide antagomir to reduce the level of miR-134 in dfECFCs under NG and HG treatment conditions (Fig 3B). The decreased level of miR-134 in HG-dfECFCs was more than that in NG-dfECFCs (a 6.8 fold decrease in HG-dfECFCs *vs*. a 2.4 fold decreased in NG-dfECFCs) because the original level of miR-134 was higher in HG-dfECFCs. Inhibition of miR-134 did not increase cell migration and tube formation in NG-dfECFCs. However, knock-down of miR-134 partially restored the reduced functionality of HG-dfECFCs (Fig 3C and S5 Fig). In addition, ECFCs from DM patients also expressed higher level of miR-134 than DF subjects. Inhibition of miR-134 in dmECFCs at 2.7 fold (Fig 3D) improved the migration and microvasculature formation activity of these cells by 3 fold and 2 fold, respectively (Fig 3E). These results suggested that miR-134, which is abundant in HG-dfECFCs and dmECFCs, hampers the angiogenic activity of ECFCs.

### Far infrared radiation rescues the functioning of ECFCs

**Fig 4:**
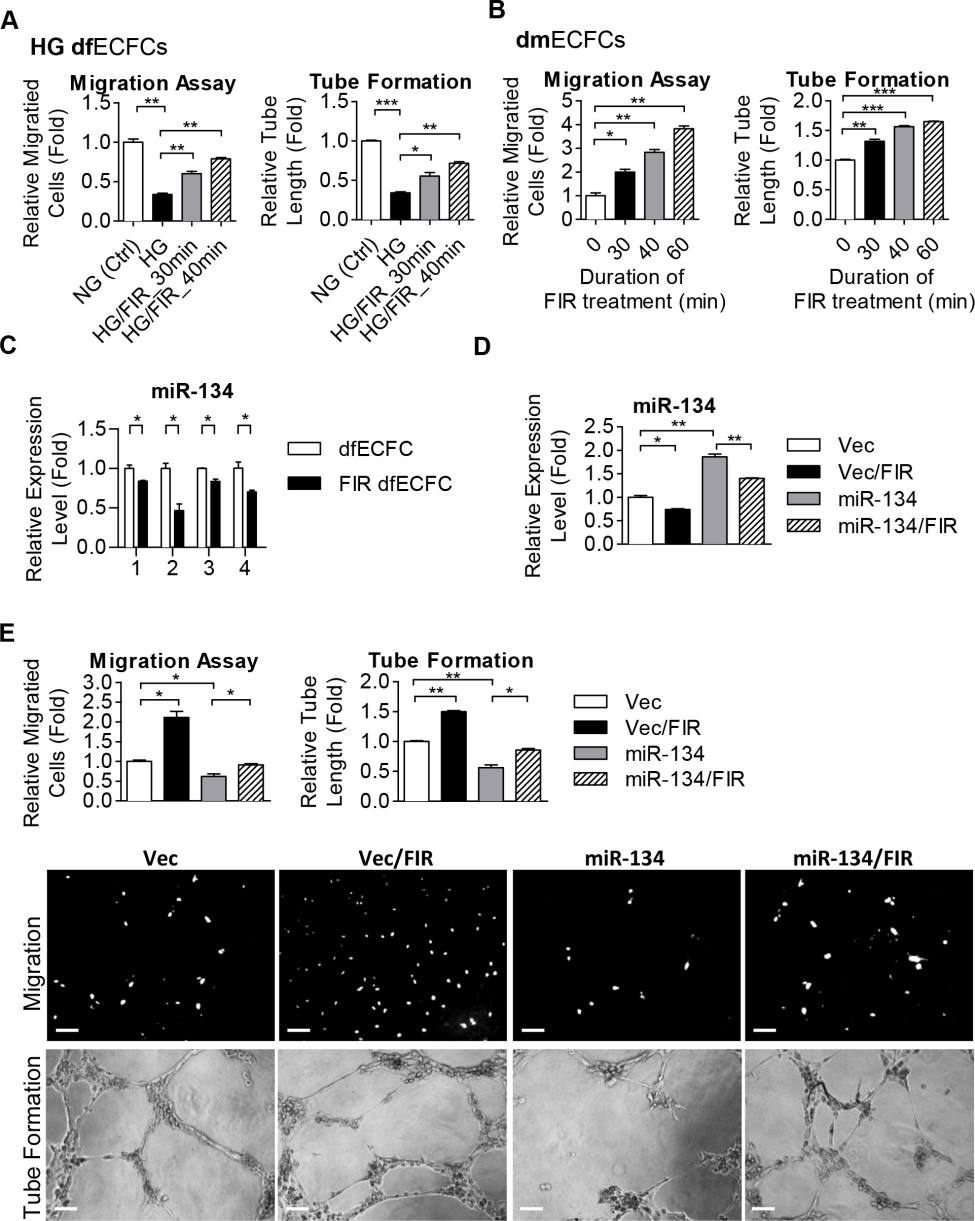
FIR treatment improves angiogenic activities of ECFCs. **(A)** Quantitative data from the Transwell cell migration assays (left) and tube formation assays (right) using HG-dfECFCs with or without FIR treatment. n = 3 independent experiments. * *p* < 0.05, ** *p* < 0.01, *** *p* < 0.001 by one-way ANOVA followed by Tukey’s post-hoc test. **(B)** Quantitative data from the Transwell cell migration assays (left) and tube assays (right) using dmECFCs treated with FIR. n = 3 independent experiments. * *p* < 0.05, ** *p* < 0.01, *** *p* < 0.001 by one-way ANOVA followed by Tukey’s post-hoc test. **(C)** The expression levels of miR-134 in dfECFCs from four individuals with or without FIR treatment. * *p* < 0.05 by one-way ANOVA followed by Tukey’s post-hoc test. **(D)** miR-134 expression in vector control and miR-134 overexpressed dfECFC with or without FIR treatment. * *p* < 0.05, ** *p* < 0.01, by one-way ANOVA followed by Tukey’s *post-hoc* test. **(E)** Representative images (lower) and quantitative data (upper) from the Transwell cell migration assays and microvascular formation assays using vector control or miR-134 overexpressed dfECFCs with or without FIR treatment. n = 3 independent experiments. * *p* < 0.05, ** *p* < 0.01 by one-way ANOVA followed Tukey’s post-hoc test. Scale bar: 50 μm.

Several previous studies have reported that FIR improves vascular endothelial function and augments angiogenesis [22, 23, 26]. We therefore explored whether FIR treatment restore the decreased activities of HG-dfECFCs and dmECFCs. HG-dfECFCs were treated with FIR for 30 or 40 minutes and the cells were then subjected to the Transwell migration and tube formation assays. Compared to HG-dfECFCs without FIR pretreatment, FIR improved HG-dfECFC motility and microvasculature structure formation by at least 1.8 and 1.6 fold, respectively (Fig 4A and S6A Fig). FIR also promoted these changes in dmECFC activities in a dose dependent manner (Fig 4B). FIR treatment of 30 to 60 minutes increased dmECFC motility by 2-fold to 3.5-fold and enhanced microvasculature formation from 1.2-fold to 1.5-fold (Fig 4B and S6B Fig). We next explored whether FIR improved the activities of HG-dfECFCs and dmECFCs via an inhibition of miR-134 expression. We detected miR-134 expression using RT-qPCR in dfECFCs from four subjects treated with or without FIR and found that the level of miR-134 was decreased after FIR treatment in four independent experiments (Fig 4C). We next used a double manipulation approach to confirm the relationship between FIR and miR-134 (Fig 4D). The decreased angiogenic activity caused by miR-134 overexpression was found to be rescued by FIR treatment (Fig 4E). In order to determine the persistent effect of FIR, HG-dfECFCs and HG-dfECFCs with FIR treatment were subjected to Transwell migration and MTT assays. The expression of miR-134 was reduced by FIR and was persistent from day1 to day 10 after FIR treatment (S7A Fig). ECFC migration ability was enhanced by FIR treatment and this effect was persistent for 10 days after FIR treatment. However, the numbers of migrated cells were gradually decreased in both cell groups because of the prolong cultivation under HG condition (S7B Fig). MTT assay performed from the indicated day after FIR treatment showed FIR slightly promoted cell proliferation to around day 10 (S7C and S7D Fig) and then cell tend to die in HG-containing media (S7E Fig, indicated with #). These findings suggested that FIR is able to reduce miR-134 to promote the functions of ECFCs and this effect could be persisted at least 10 days.

### Microarray analyses reveal that NRIP1 was the miR-134 target

**Fig 5:**
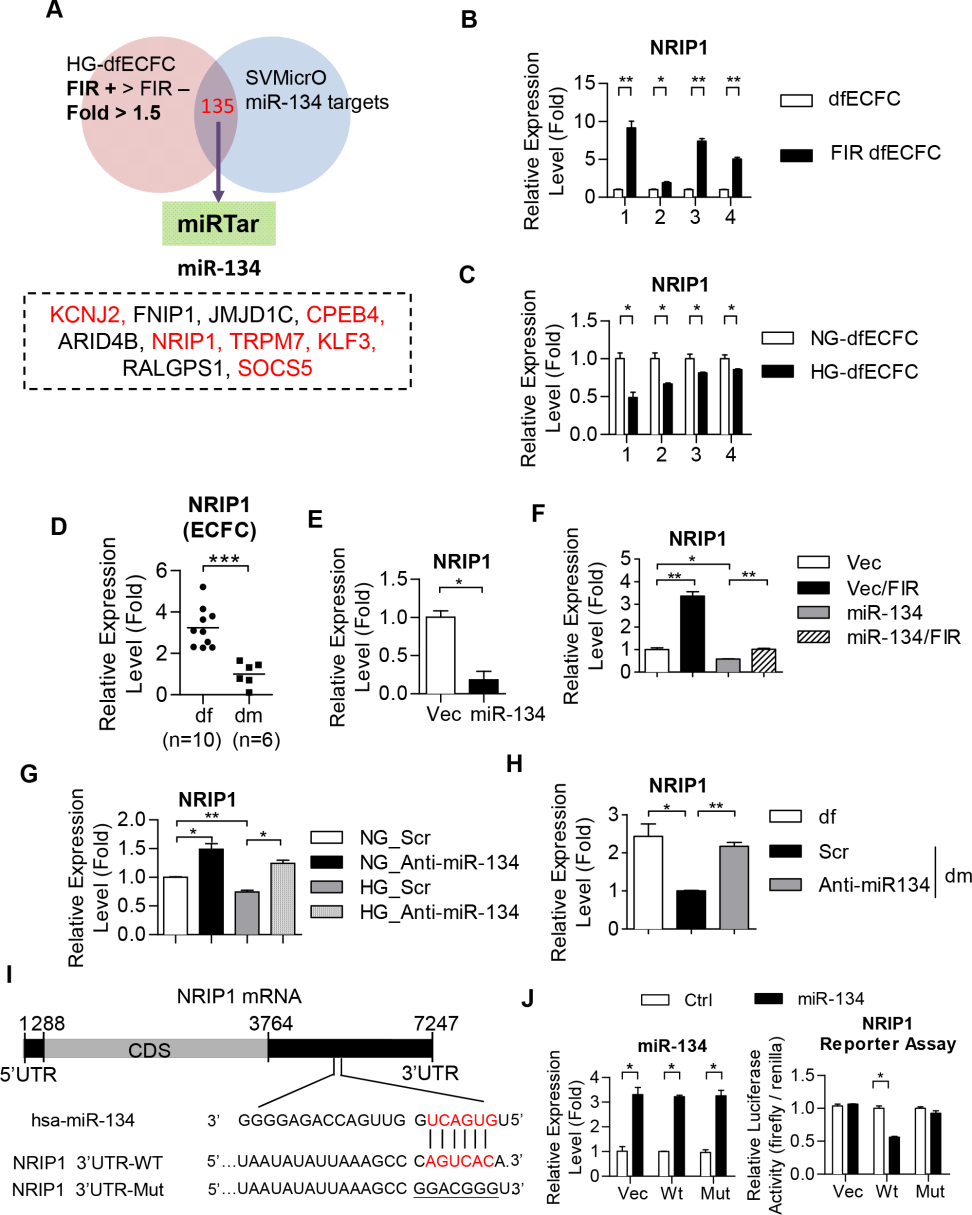
NRIP1 is a direct target of miR-134. **(A)** A Venn diagram shows the principle used to filter the target genes of miR-134. **(B, C)** Validation of putative miR-134 downstream targets in dfECFCs under FIR (B) and high glucose (C) treatment using RT-qPCR. * *p* < 0.05, ** *p* < 0.01 by one-way ANOVA followed by Tukey’s post-hoc test. **(D)** Relative expression level of NRIP1 in dfECFCs and dmECFCs. *** *p* < 0.001 by Mann-Whitney U test **(E, F)** Validation of NRIP1 expression in miR-134 overexpressed dfECFCs (E) and in combination with FIR treatment (F). n = 3 independent experiments. * *p* < 0.05 (E) by Mann-Whitney U test, * *p* < 0.05, ** *p* < 0.01 (F) by one-way ANOVA followed by Tukey’s post-hoc test. **(G, H)** The expression levels of NRIP1 in HG-dfECFCs or dmECFCs treated with scramble control or miR-134 antagomir. n = 3 independent experiments. * *p* < 0.05, ** *p* < 0.01 one-way ANOVA followed by Tukey’s post-hoc test. **(I)** Structure of the NRIP1 transcript and the predicted miR-134 binding site on the NRIP1-3’UTR. **(J)** The relative luciferase activities of the vector control or miR-134 overexpressed 293T cells co-transfected with the wild type or mutant NRIP1 3’UTR reporter plasmids (right panels). Luciferase activity was normalized against the vector control group and are presented as the mean + SD. The expression levels of each miRNAs as detected by RT-qPCR (left panels). n = 3 independent experiments. * *p* < 0.05 by one-way ANOVA followed by Tukey’s post-hoc test. Scr, scramble control; Vec, plasmid control; Wt, wild type 3’UTR of NRIP1; Mut, mutated 3’UTR of NRIP1.

In order to determine the mechanism involved in regulating ECFC activity, we identified genes involved in FIR treatment by microarray analysis (GSE37044) [37]. We focused on genes that were enhanced by FIR (Fig 5A) and predicted targets of miR-134 using SVMicrO. The 135 genes were subject to miRTar to narrow down the putative targets of miR-134 and 35 putative targets were identified (Fig 5A). Among the top 10 enhanced genes that are putative miR-134 targets, 6 genes were reported to be involved in diabetes or angiogenesis. These six genes, namely potassium channel, inwardly rectifying subfamily J, member2 (KCNJ2) [38], cytoplasmic polyadenylation element binding protein 4 (CPEB4) [39], nuclear receptor-interacting protein 1 (NRIP1), transient receptor potential cation channel, subfamily M, member 7 (TRPM7) [40, 41], Kruppel-like factor 3 (KLF3) [42] and suppressor of cytokine signaling 5 (SOCS5) [43] (Fig 5A). These genes were then validated using RT-qPCR. Isolated dfECFCs from four individuals were treated with FIR to determine the expression of the six targets. We found that only NRIP1was increased in all four FIR treated ECFC samples compared to non-FIR treated cells (Fig 5B and S8A Fig). In addition, NRIP1 showed a lower expression level in HG-dfECFCs compared to NG-dfECFCs (Fig 5C). NRIP1 was also reduced in ECFCs from clinical DM patients (Fig 5D). These results suggest that FIR treatment is able to reduce miR-134 expression, which in turn enhances the downstream expression level of NRIP1. In contrast, DM or HG treatment leads to miR-134 upregulation in ECFCs and a decrease in NRIP1 expression.

We further investigated whether NIRP1 is a downstream target of miR-134. Overexpression of miR-134 in dfECFCs (S8B Fig) results in a reduction of 90% in NRIP1 expression (Fig 5E). Combining FIR treatment with miR-134 overexpressed ECFCs reversed this decrease in NRIP1 expression and results in a similar level of expression to that found in the vector control (Fig 5F). Furthermore, NRIP1 expression not only significantly increased in both antagomir treated NG-dfECFCs and HG-dfECFCs (Fig 5G and S8C Fig), but also in dmECFCs with miR-134 antagomir treatment (Fig 5H). We next used a 3’UTR luciferase reporter assay to clarify the direct targeting of NRIP1 by miR-134. The putative miR-134 binding region within the 3’UTR of NRIP1 is shown in Fig 5I. miR-134 overexpression repressed the luciferase activity of the wild type NRIP1 3’UTR, but this reduction was reversed using a mutated NRIP1’s 3’UTR (Fig 5J). These findings suggested that miR-134 directly targets the 3’ UTR of NRIP1 to attenuate angiogenic activity of ECFCs *in vitro*.

### NRIP1 is involved in various ECFC activities and is crucial for miR-134 functionality

**Fig 6:**
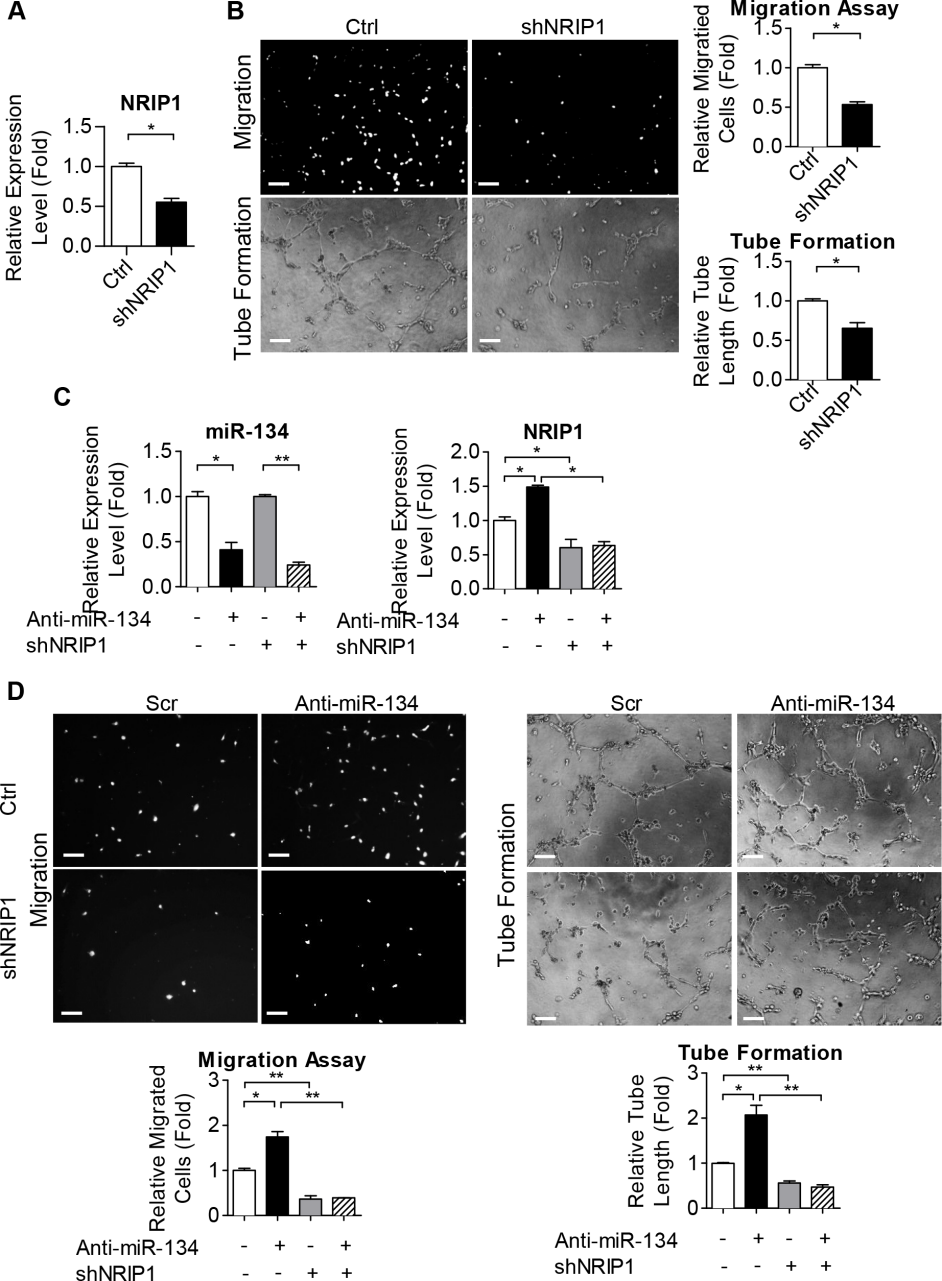
NRIP1 is involved in ECFC activity and is crucial for miR-134 functionality. **(A)** Validation of NRIP1 in dfECFCs infected with NRIP1 shRNA. * *p* < 0.05 by Mann-Whitney U test. **(B)** Representative images (left) and quantitative data (right) from the Transwell migration assays (upper) and microvascular formation assays (lower) using ECFCs infected with control or NRIP1 shRNA plasmids. n = 3 independent experiments. * *p* < 0.05 by Mann-Whitney U test. Scale bar: 50 μm. **(C)** The expression levels of miR-134 and NRIP1 in dfECFCs treated with miR-134 antagomir and NRIP1 shRNA as a double manipulation. n = 3 independent experiments. * *p* < 0.05, ** *p* < 0.01 by one-way ANOVA followed by Tukey’s post-hoc test. **(D)** Representative images (upper) and quantitative data (lower) of the Transwell cell migration assays (left) and tube formation assays (right) using dfECFCs with miR-134 antagomir and NRIP1 shRNA as a double manipulation. n = 3 independent experiments. * *p* < 0.05, ** *p* < 0.01 by one-way ANOVA followed by Tukey’s post-hoc test. Scale bar: 50 μm. Ctrl: plasmid control, shNRIP1: NRIP1 shRNA, Anti-miR-134: miR-134 antagomir.

In order to explore the role of NRIP1 in ECFCs, we used shRNA to knockdown NRIP1 in dfECFCs (Fig 6A) and this led to a 2.5-fold reduction in cell motility and a 2-fold reduction in microvasculature structure formation (Fig 6B). We further used an anti-miR-134 antagomir combined with shNRIP1 to clarify the interaction between miR-134 and NRIP1 (Fig 6C). The increase in cell migration and tube formation activities caused by miR-134 inhibition was reduced by NRIP1 knockdown in dfECFCs (Fig 6D). These results suggest that NRIP1 is a direct target of miR-134 and is involved in ECFCs functioning. We also determined the relation between FIR and NRIP1 by combining FIR treatment in NRIP1 knockdown or control HG-dfECFCs. The expression of NRIP1 was determined to clarify the knockdown efficiency (S9A Fig). The FIR induced microvasculature formation in plasmid control HG-dfECFC was suppressed by inhibition of NRIP1 (S9B and S9C Fig). The results suggest the FIR modulates the functions of ECFCs through miR-134-NRIP1 axis.

### FIR induces ECFC activation and promotes blood flow recovery using a mouse ischemic limb model

**Fig 7:**
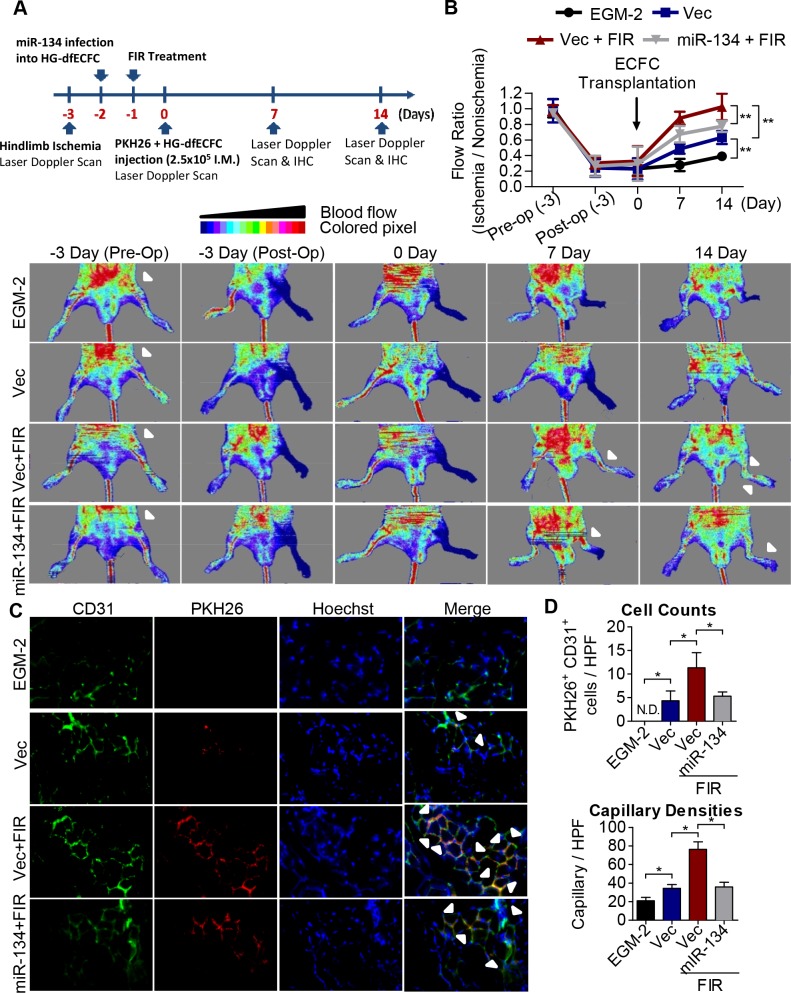
FIR induces ECFC activation and improves blood perfusion using an ischemic hindlimb model. **(A)** Schematic representation of experimental design. **(B)** Upper: quantitative analysis of blood flow expressed as perfusion ratio of the ischemic to the non-operated contralateral hindlimb. n = 6, ** *p* < 0.01 by one-way ANOVA followed by Tukey’s post-hoc test. Lower: representative images of mouse ventral side during the measurement of hindlimb blood flow by laser Doppler before operation (Pre-Op), immediately after hindlimb ischemia surgery (Post-Op), and 2 weeks after intramuscular injection of culture medium (EGM2), HG-dfECFCs (Vec), HG-dfECFCs with FIR treatment (Vec + FIR), and miR-134 overexpressed HG-dfECFCs with FIR treatment (miR-134 + FIR). **(C)** Immunofluorescence staining of the tissue from nude mice 7 days after injection with PKH-26-labeled HG-dfECFCs. The capillaries in the limb muscles were visualized by anti-CD31 immunostaining (green), and injected human ECFCs were monitored by PKH-26 fluorescence (red). In addition there was Hoechst nuclear staining of the live cells (blue). **(D)** Quantitative analysis of CD31^+^/PKH-26^+^ double-positive cells and capillary densities in limb muscle of mice hindlimb ischemia region. HPF: high power field; N.D.: not detectable; * *p* < 0.05 by one-way ANOVA followed by Tukey’s post-hoc test.

We next evaluated the therapeutic potential of FIR in terms of the *in vivo* angiogenic activity of HG-dfECFCs. Nude mice received femoral artery excision surgery to develop limb ischemia three days before ECFC injection. At 48 hours prior to transplantation, we overexpressed a vector control and miR-134 in the HG-dfECFCs. Furthermore, the ECFCs were treated with FIR one day before ECFC injection (Fig 7A). At day 0 after injection, miR-134 and NRIP1 were detected using RT-qPCR to confirm that miR-134 expression was reduced and NRIP1 expression was increased by FIR (S10A Fig). A reduction in blood flow was observed in left leg after surgery (day 3) until ECFC injection (day 0) (Fig 7B). HG-dfECFCs with different treatment were injected intramuscularly at the ischemic hindlimb from distal to the arterial occlusion site 3 days after surgery and the blood flow was then measured by Laser Doppler perfusion imaging (LDPI) at day 7 and day 14. Although blood flow in the ischemic leg had recovered slightly in the medium control group (the “EGM2” group) on day 14 (Fig 7B), HG-dfECFCs transplantation (the “Vec” group) improved the blood flow ratio from 0.29 on day 0 to 0.6 on day 7 and 0.73 on day 14. In addition, the improvement of blood flow was more significant in FIR-treated HG-dfECFCs (the “Vec + FIR” group) with an increase in the ratio to 0.88 on day 7 and 1.0 on day 14, which is close to the pre-operation condition. Overexpression of miR-134 in the FIR-treated HG-dfECFCs (the “miR-134 + FIR”) reduced the flow ratio to 0.67 on day 7 and 0.77 on day 14 (Fig 7B).

To investigate the effects of FIR on the homing and angiogenic activities of the injected ECFCs, we conducted immunofluorescence staining of the mouse limb tissues at day 7 (Fig 7C) and 14 (S10B Fig) after HG-dfECFCs injection. Capillary density was examined as an index of angiogenesis using immunostaining with CD31 antibody on day 7 (green, Fig 7C) and day 14 (S10B Fig). The injected human HG-dfECFCs pre-labeled with PKH-26 fluorescence indicated that homing and involvement of ECFCs in microvascular repair did occur (red, Fig 7C). Mice in the FIR treated HG-dfECFCs group (the “Vec + FIR” group) presented with more CD31^+^/PKH-26^+^ double-positive cells (white arrowheads) in the capillaries of the limb muscle than the Vec and miR-134/FIR groups (Fig 7C; quantitative data in Fig 7D). In addition, the capillary densities of the limb muscle were also higher in mice treated with FIR than the Vec and miR-134/FIR groups (Fig 7D). The findings obtained using the ischemia limb model suggested that FIR is able to restore the angiogenic activity of HG-dfECFCs and improve vascular repair in mice *in vivo*.

## Discussion

Type 2 diabetes mellitus is a chronic metabolic disease and is a growing public health issue in the world [44]. T2D is associated with insulin resistance, hyperglycemia and dyslipidemia and is a risk factor for cardiovascular diseases [45]. In addition, patients with DM are predisposed to endothelial dysfunction and several vascular complications [46, 47]. Several mechanisms of endothelial dysfunction in DM have been identified including alteration in signaling related to eNOS activation, increased oxidative stress, activation of inflammatory processes and impaired mitochondrial functioning [48]. In this study, we focus on the effects of high glucose on a subset of endothelial progenitor cells, namely ECFCs, which display clonal proliferative potential and *in vivo* vascular formation activity [49–51]. Several studies have suggested that ECFCs are a potential cell source for regenerative medicine because of the ability of ECFCs to home onto ischemic regions, to increase microvascular density, to promote vascular repair and to help recovery of blood flow [52–55]. Furthermore, ECFCs with high glucose treatment or isolated from GDM pregnancies have a slower cell proliferation rate, impaired cell migration ability and reduced tube formation ability [9, 10]. In this study, ECFCs treated with HG also show similar results to those presented in a previous study. In addition, ECFCs treated with HG/LGF show poorer ECFC functionalities than the HG group (Fig 1E and 1F) and this result is reasonable since the HG/LGF group mimics the physical reaction of the poorest progression of DM patients. We have also identified that HG increases three miRNAs in ECFCs, namely miR-370, miR-183-5p and miR-134. However, only miR-370 and miR-134 show enhanced expression levels in dmECFCs. Overexpression of miR-134, but not of miR-370, in dfECFCs results in decreased cell migration and poorer tube formation ability (Fig 3A). Although dmECFCs show higher level of miR-370 than dfECFCs, it seems that miR-370 may also regulate other cellular mechanisms in DM. According to previous studies, miR-370 is a lipid metabolism-related miRNA [56] and the increased miR-370 expression in hyperlipidemia patients has been positively correlated with the severity of coronary artery disease [57]. Several genes, including sterol-regulatory element binding protein 1c (SREBP-1c), enzymes diacylglycerol acyltransferase-2 (DGAT2), fatty acid synthase (FAS) and acyl-CoA carboxylase 1 (ACC1), that are all involved in regulation of fatty acid and triglyceride biosynthesis, are increased by miR-370 in HepG2 cells. miR-370 also targets carnitine palmitoyltransferase 1α (Cpt1α), which is a mitochondria enzyme and is involved in transportation of long-chain fatty acid for energy production, resulting in a decreased rate of beta oxidation [58]. The accumulation of hepatic fatty acid has been shown to be associated with insulin resistance and metabolic syndrome.

Previous studies have suggested that miR-134 overexpression promotes neuronal cell death and apoptosis by regulating response element-binding protein (CREB) signaling and targeting heat shock protein A12B (HSPA12B) [59, 60]. In retinal ganglion cells (RGCs), miR-134 is increased by H_2_O_2_ treatment and lead to RGC apoptosis [61]. However, miR-134 overexpression in ECFCs did not alter their cell proliferation rate based on the results from the MTT assays or result in cell cycle arrest based on the flow cytometry analysis. Our results and previous studies both show that decreased ECFC proliferation in DM and in high glucose condition, but it seems that this occurs in a miR-134-independent manner. In contrast, miR-134 was first identified as an angiogenic modulator that decreases ECFC migration and microvascular formation. Hyperglycemia damages endothelial cells by several mechanisms including increased reactive oxygen species, decreased nitric oxide (NO) bioavailability, augmentation of glycation end products (AGEs) and an over-activated hexosamine pathway [62]. HG-treated ECFCs have been shown to have decreased levels of phosphorylated endothelial nitric oxide synthase (eNOS) and bioavailable NO [9]. However, whether hyperglycemia also impairs ECFCs via these mechanisms in endothelial cells is still unknown. Furthermore, whether reactive nitrogen species (RNS) or reactive oxygen species (ROS) participate in the HG-induced miR-134 modulation of ECFCs motility and tube formation need further investigation.

We explored how NRIP1 regulates ECFC activities and is the target of miR-134 in this study. NRIP1 controls several physiological responses via binding with other transcription factors and histone modifying enzymes such that it can act as either a coactivator or a corepressor [63]. A previous study has reported that NRIP1 expression is higher in diabetes patients and correlates with inflammatory cytokines in plasma and peripheral blood mononuclear cells (PBMCs) [64]. In human skeletal muscle, insulin triggers the mitogen-activated protein kinase (MAPK) and phosphoinositide 3-kinase (PI3K)-Akt pathways to phosphorylate TBC1 domain family, member 4 (TBC1D4, also called AS160), a Rab-GTPase-activating protein (Rab-GAP) [65]. This then results in the translocation of glucose transporter type 4 (GLUT4) storage vesicles to plasma membrane and increased glucose uptake. However, cytoplasmic NRIP1 impairs GLUT4 trafficking via interacting with AS160, which reduces the level of Akt-mediated phosphorylation [66]. These results suggest that NRIP1 acts as negative regulator in relation to glucose uptake and thus would seem likely to contribute to various metabolism diseases. Further investigation is needed to address how NRIP1 modulates ECFC activities and if any factors that bind with NRIP1 are able to exert angiogenic activities.

FIR is an electromagnetic radiation with wavelengths from 5.6–1000 um. FIR has been applied to a variety of fields such as food preservation [67] and health improvement, including increased growth, better sleep [20], better microcirculation [25, 68], improved wound healing [69] and decreased chronic pain [21]. In this study, FIR treated HG-dfECFCs improved blood flow recovery in hindlimb ischemia model, which is a simpler model of peripheral arterial disease and is useful for testing new therapies. Notably, the variability in the result of Laser Doppler results of blood flow may arise from inconsistently positioned leg and different sizes regions of interest. We minimized the variability in data analysis by fixing selected size in interested region with moor LDI software. A previous study has shown that FIR activates p38 and extracellular signal-regulated kinase (ERK), rather than Akt and c-Jun N-terminal protein kinases (JNK), to promote angiogenic activities after 30 minutes of FIR radiation [70]. In renal cell carcinoma cells, miR-134 also modulates the MAPK/ERK pathway and acts as a tumor suppressor [71]. However, whether the above-mentioned signaling pathways are affected by miR-134 regulation under FIR treatment and the detailed interactions that occur between miR-134 and these pathways need further investigation. Nevertheless, miR-134 seems to be a marker for determining the effectiveness of FIR treatment.

Endothelial nitric oxide synthase (eNOS), an enzyme that generates nitric oxide (NO), is crucial for normal endothelial functions, including modulation of vascular dilator tone and protection of vessels from injurious consequences. Dysregulation of eNOS activities and diminish release of NO contribute to several physical diseases. Repeat FIR sauna treatment increases eNOS, augments angiogenesis [26, 72] and attenuates cardiac remodeling after myocardial infarction (MI) [73]. However, HMVECs treated with FIR emitter do not show an increase in phosphorylated eNOS. Based on our microarray datasets, there is also no significant enhancement of eNOS in ECFCs treated with FIR when comparing to non-FIR treatment. The discrepancy may be due to differences in the FIR devices used or to differences in the duration of FIR treatment; these differences also need further investigation.

In addition to hyperglycemia, elevated free fatty acids (FFA) and dysregulated hormones are common issues in diabetes and contribute to endothelial dysfunctions. Comparing the obese nondiabetic subjects with T2D patients, saturated fatty acids (SFA), but not polyunsaturated fatty acids (PUFA), increase significantly in the plasma of DM patients [74]. Several studies have showed that FFAs decrease NOS activity and reduce NO production in endothelial cells by impairing PLC-medicated Ca^2+^ signaling or inhibiting insulin-induced insulin receptor substrate 1 (IRS-1) and Akt pathway [75, 76]. Saturated fatty acid, especially palmitic acid (PA), is the most studied fatty acid in ECFCs. PA activates p38 MAPK, JNK or Akt to increase apoptosis or decrease angiogenic activities of ECFCs in a dose-dependent manner [77, 78]. However, the effects of PA are different depend on the concentration. Only diabetic PA, but not physiologic PA, impairs ECFC proliferation through decreasing phosphorylation form of STAT5 [79]. Whether other saturated, monounsaturated or polyunsaturated FAs, regulate ECFC angiogenic activities remains unclear.

Stress response is a reaction to deal with anything that disrupts the body homeostasis. Chronic stress induces hormones, such as glucocorticoids (GC) and catecholamines, which not only regulate angiogenic activities [80, 81], but also lead to various manifestations of metabolic syndrome, including diabetes [82]. ECFCs isolated from GC-induced avascular osteonecrosis of the femoral head show decreased cell proliferation and tube formation capacities when compared to healthy subjects [83]. In addition, dopamine induces endocytosis of VEGF receptor 2 and results in preventing VEGFA-induced angiogenesis [84]. Dopamine also reduces VEGFA-induced mobilization of bone marrow CD45^-^CD34^+^VEGFR2^+^ cells to peripheral blood and results in restrained tumor growth [85]. However, norepinephrine shows opposite effect on mobilization of CD45^-^CD34^+^VEGFR2^+^ cells in mice with hindlimb ischemia [86]. The consequence of FFA and stress-induced hormones to ECFCs and mature endothelial cells varies considerably depending on the cellular milieu and need further studies.

## Conclusions

In this study, we are the first to provide the evidence that FIR treatment suppresses miR-134, which is induced under HG condition. The reduction of miR-134 results in increasing NRIP1 level and thus improves the angiogenic activities of ECFCs *in vitro*. The FIR treated HG-dfECFCs also show that this treatment has *in vivo* therapeutic potential since it improves blood flow perfusion using a hindlimb ischemic model. Our findings provide a biological effect for FIR and suggest that FIR treatment may be able to improve the progression of DM patients.

## Supporting Information

S1 Fig Expression of indicated molecules in ECFCs using flow cytometry analyses. **(A)** FACS analysis of the surface antigens present on dfECFCs and dmECFCs. **(B)** The cell surface markers expressed on NG-dfECFCs, HG-dfECFCs, LGF-dfECFCs and HG/LGF-dfECFCs.
(TIF)

S2 Fig The cell cycle of ECFCs and the angiogenic activities of HUVECs under NG, HG, LGF and HG/LGF conditions. **(A)** Cell cycle analysis of dfECFCs under normal culture (NG), high glucose (HG), low growth factor (LGF) and HG/LGF conditions using flow cytometry. **(B)** Representative images (upper) and quantitative data (lower) for the cell migration assays and tube formation assays using HUVECs. ** *p* < 0.01, *** *p* < 0.001 by one-way ANOVA followed by Tukey’s post-hoc test. Scale bar: 50 μm.
(TIF)

S3 Fig RT-qPCR results showing the expression levels of 7 miRNAs present in microarrays of NG-dfECFCs and HG-dfECFCs. * *p* < 0.05, ** *p* < 0.01 by one-way ANOVA followed by Tukey’s post-hoc test.
(TIF)

S4 Fig The effect of miR-370 and miR-134 overexpression on dfECFCs or HUVECs. **(A)** dfECFCs were infected with lenti-miR-370 and lenti-miR-134. The expression levels of miRNA are quantified by RT-qPCR. ** *p* < 0.01 by one-way ANOVA followed by Tukey’s post-hoc test. **(B)** Cell proliferation rate in dfECFCs with miR-370 and miR-134 overexpression. **(C)** Flow cytometry analysis of the cell cycles of miR-370 and miR-134 overexpressed dfECFCs. **(D)** Overexpression of miR-370 and miR-134 in HUVECs and the expression levels of these miRNAs as quantified by RT-qPCR. * *p* < 0.05, ** *p* < 0.01 by one-way ANOVA followed by Tukey’s post-hoc test. **(E)** Representative images (lower) and quantitative data (upper) for the Transwell migration assays and tube formation assays using HUVECs with miR-370 and miR-134 overexpression. * *p* < 0.05, ** *p* < 0.01 by one-way ANOVA followed by Tukey’s post-hoc test. Scale bar: 50 μm.
(TIF)

S5 Fig Representative images of the cell migration assays and tube formation assays of scramble control or miR-134 antagomir treated NG-dfECFCs and HG-dfECFCs. Scale bar: 50 μm.
(TIF)

S6 Fig Representative images of the cell migration assays and tube formation assays of HG-dfECFCs (A) and dmECFCs (B) after FIR treatment. Scale bar: 50 μm.
(TIF)

S7 Fig The persistent effect of FIR. **(A)** The expression of miR-134 at indicated days after FIR treatment in HG-dfECFCs. * *p* < 0.05, ** *p* < 0.01 by one-way ANOVA followed by Tukey’s post-hoc test. (**B)** The quantative data of migrated cell numbers at indicated days after FIR treatment. * *p* < 0.05, ** *p* < 0.01 by one-way ANOVA followed by Tukey’s post-hoc test. **(C~E)** MTT cell proliferation assay performed after 3, 8 and 10 days after FIR treatment. * *p* < 0.05 by Mann-Whitney U test.
(TIF)

S8 Fig Validation of the putative downstream targets of miR-134. **(A)** Validation of the putative miR-134 target genes by RT-qPCR in dfECFCs with or without FIR treatment. * *p* < 0.05, ** *p* < 0.01, *** *p* < 0.001 by one-way ANOVA followed by Tukey’s post-hoc test. **(B, C)** The expression level of miR-134 in miR-134 overexpressed ECFCs (B) and miR-134 antagomir treated ECFCs (C). * *p* < 0.05 (B) by Mann-Whitney U test, ** *p* < 0.01, *** *p* < 0.001 (C) by one-way ANOVA followed by Tukey’s post-hoc test.
(TIF)

S9 Fig Tube formation ability in NRIP1 knockdown HG-dfECFCs or plasmid control HG-dfECFCs either combined FIR treatment. **(A)** The expression levels of NRIP1 in each group of ECFCs. * *p* < 0.05, ** *p* < 0.01 by one-way ANOVA followed by Tukey’s post-hoc test. **(B)** The quantitative data of tube formation assay. * *p* < 0.05 by one-way ANOVA followed by Tukey’s post-hoc test. **(C)** Representative images of the microvascular formation assays in each group cells. Scale bar: 50 μm.
(TIF)

S10 Fig Transplantation of FIR treated HG-dfECFCs improves blood perfusion in ischemic hindlimb regions. **(A)** Validation of the expression levels of miR-134 and NRIP1 in each group of ECFCs by RT-qPCR on day 0. * p < 0.05 by one-way ANOVA followed by Tukey’s post-hoc test. **(B)** Immunofluorescence staining of nude mice tissue samples on day 14 after injection with HG-dfECFCs. Capillaries in the ischemic muscles were visualized by anti-CD31 immunostaining (green), Hoechst: nuclear staining of live cells (blue).
(TIF)

S1 Table qPCR Primer Sequences. (XLSX)

S2 Table Cloning Primer Sequences. (XLSX)
