# Supplementary figures and images for: MicroRNA-134 Contributes to Glucose-Induced Endothelial Cell Dysfunction and This Effect Can Be Reversed by Far-Infrared Irradiation
Source: PLoS One. 2016 Jan 22;11(1):e0147067. doi: 10.1371/journal.pone.0147067 (PMC4723308; doi:10.1371/journal.pone.0147067)

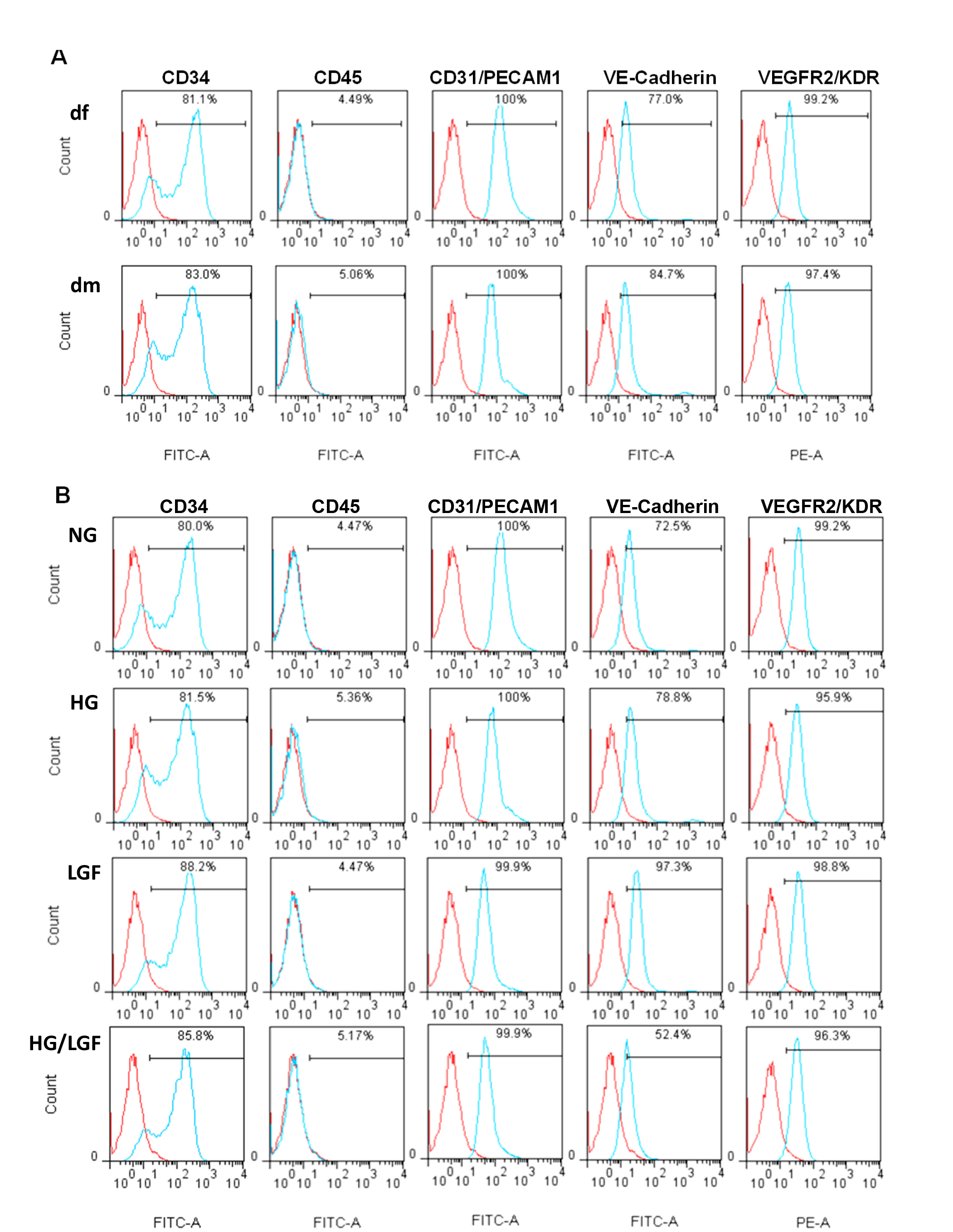

Supplement: S1 Fig — (A) FACS analysis of the surface antigens present on dfECFCs and dmECFCs. (B) The cell surface markers expressed on NG-dfECFCs, HG-dfECFCs, LGF-dfECFCs and HG/LGF-dfECFCs. (TIF) [file pone.0147067.s001.tif]

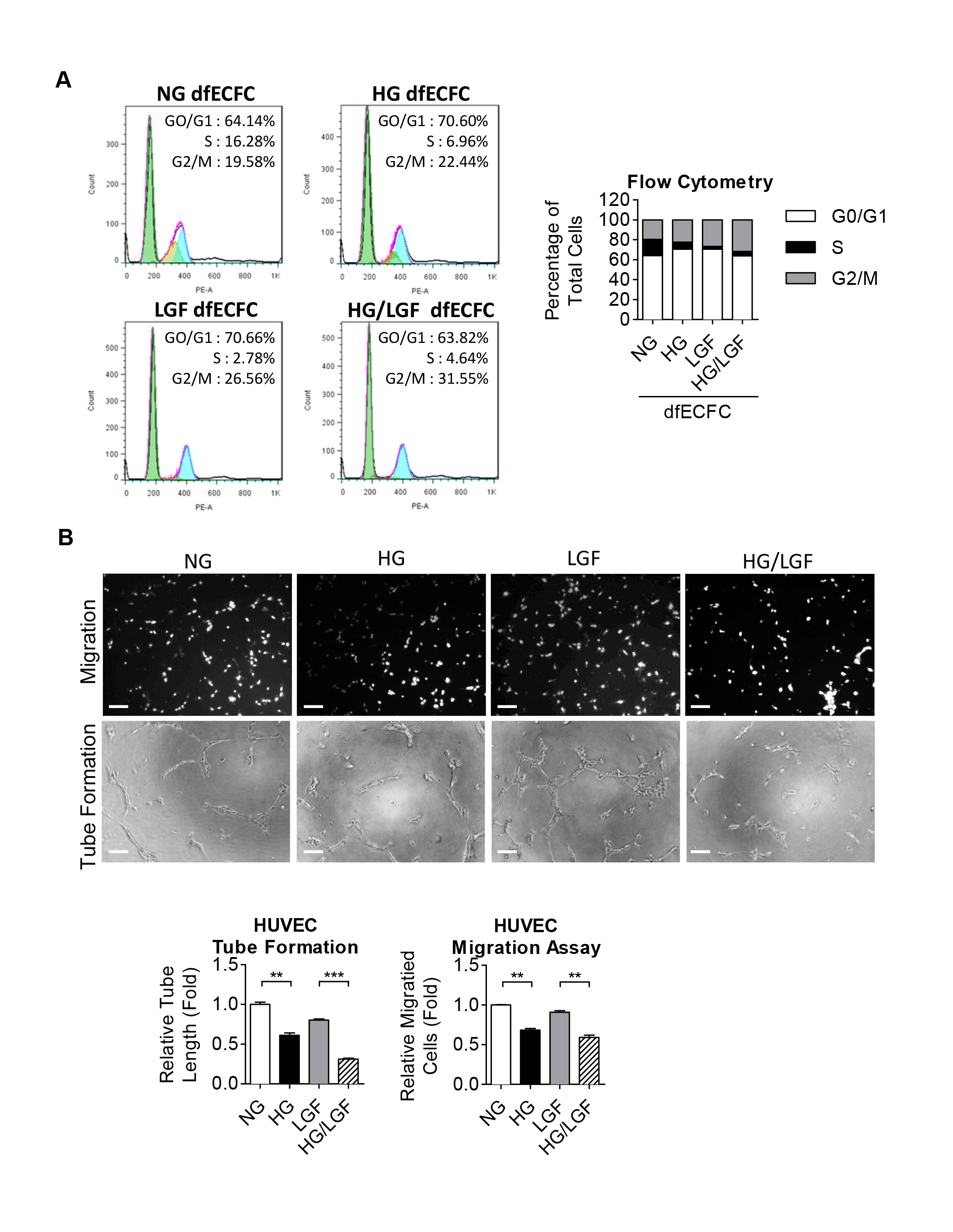

Supplement: S2 Fig — (A) Cell cycle analysis of dfECFCs under normal culture (NG), high glucose (HG), low growth factor (LGF) and HG/LGF conditions using flow cytometry. (B) Representative images (upper) and quantitative data (lower) for the cell migration assays and tube formation assays using HUVECs. ** p < 0.01, *** p < 0.001 by one-way ANOVA followed by Tukey’s post-hoc test. Scale bar: 50 μm. (TIF) [file pone.0147067.s002.tif]

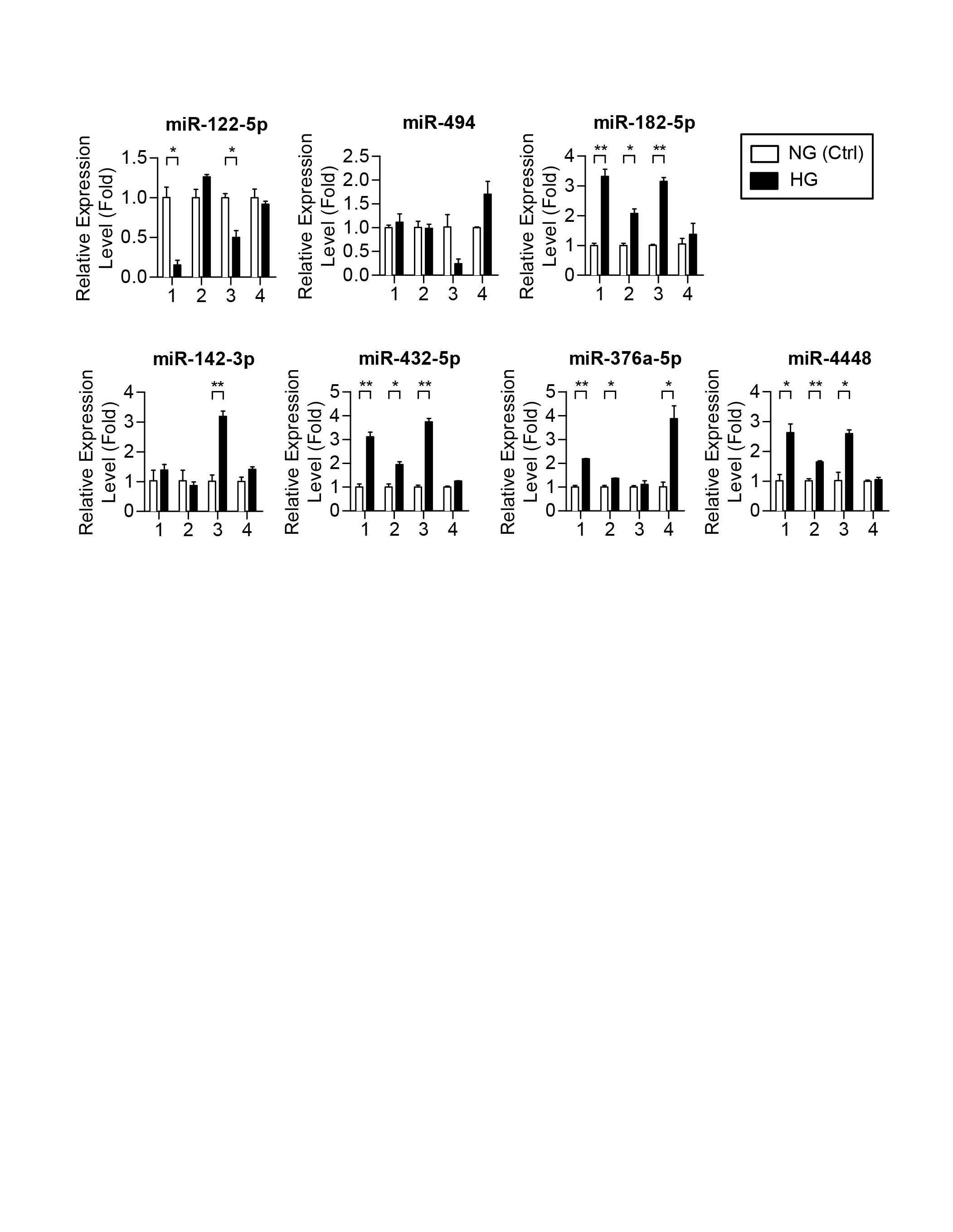

Supplement: S3 Fig — * p < 0.05, ** p < 0.01 by one-way ANOVA followed by Tukey’s post-hoc test. (TIF) [file pone.0147067.s003.tif]

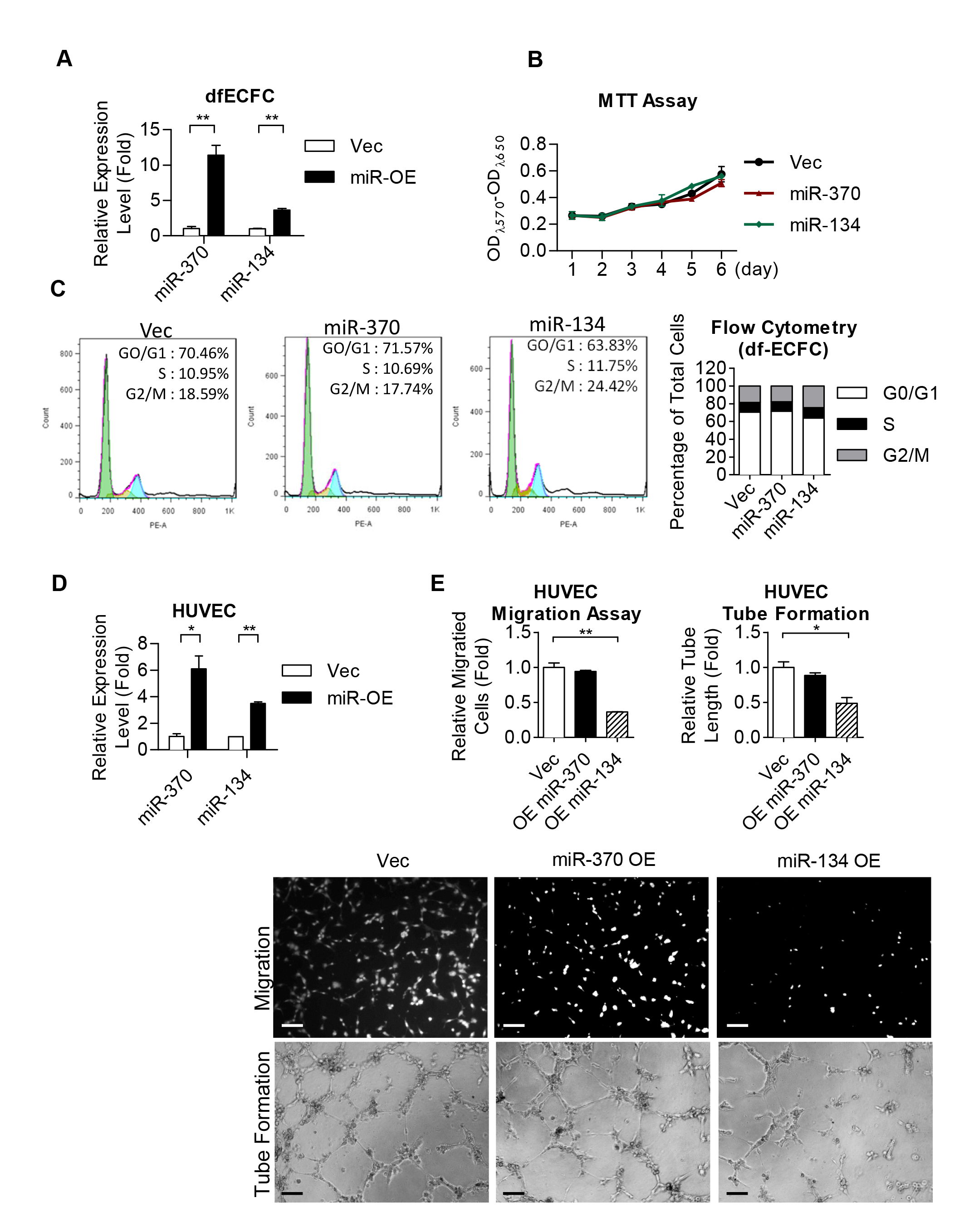

Supplement: S4 Fig — (A) dfECFCs were infected with lenti-miR-370 and lenti-miR-134. The expression levels of miRNA are quantified by RT-qPCR. ** p < 0.01 by one-way ANOVA followed by Tukey’s post-hoc test. (B) Cell proliferation rate in dfECFCs with miR-370 and miR-134 overexpression. (C) Flow cytometry analysis of the cell cycles of miR-370 and miR-134 overexpressed dfECFCs. (D) Overexpression of miR-370 and miR-134 in HUVECs and the expression levels of these miRNAs as quantified by RT-qPCR. * p < 0.05, ** p < 0.01 by one-way ANOVA followed by Tukey’s post-hoc test. (E) Representative images (lower) and quantitative data (upper) for the Transwell migration assays and tube formation assays using HUVECs with miR-370 and miR-134 overexpression. * p < 0.05, ** p < 0.01 by one-way ANOVA followed by Tukey’s post-hoc test. Scale bar: 50 μm. (TIF) [file pone.0147067.s004.tif]

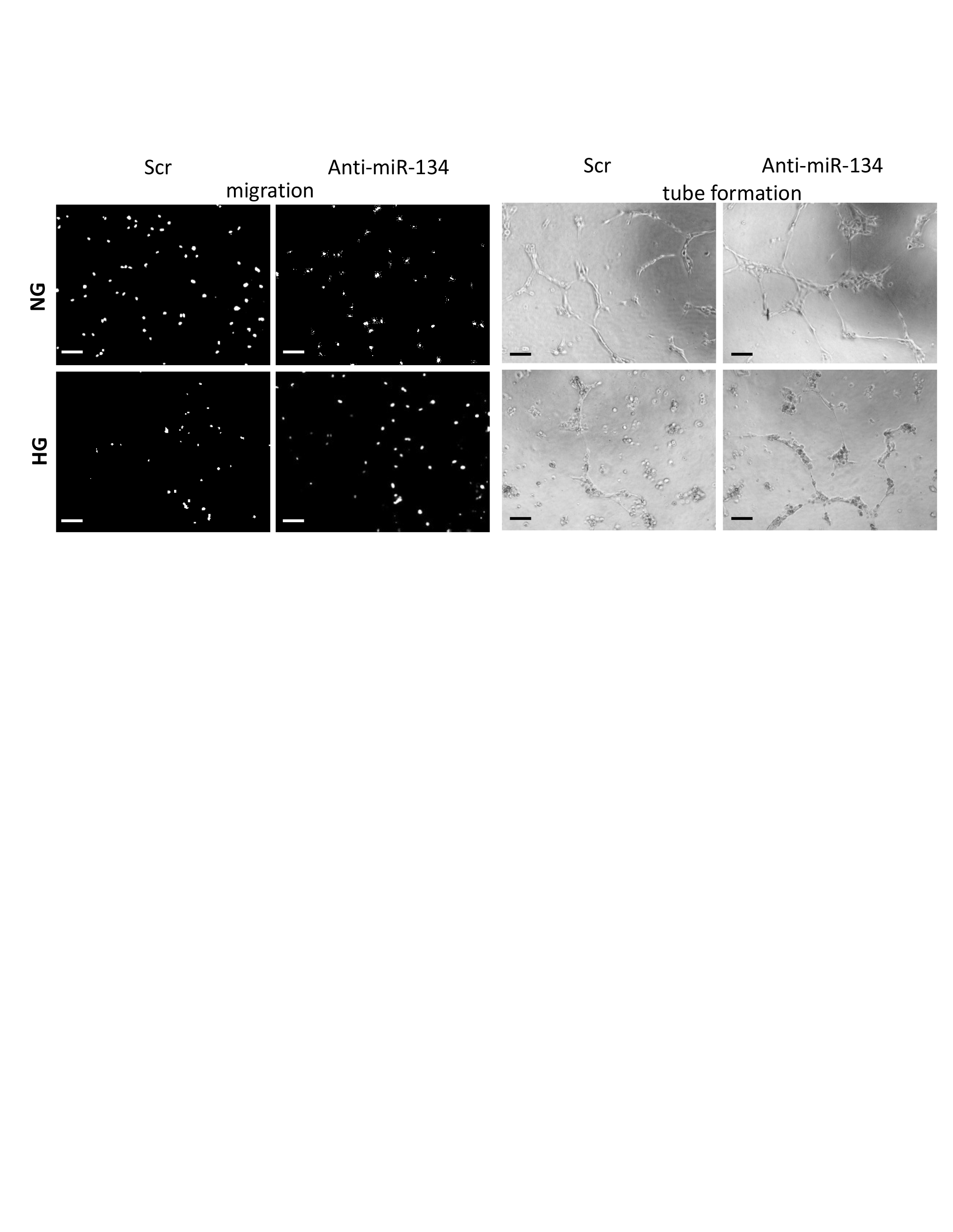

Supplement: S5 Fig — Scale bar: 50 μm. (TIF) [file pone.0147067.s005.tif]

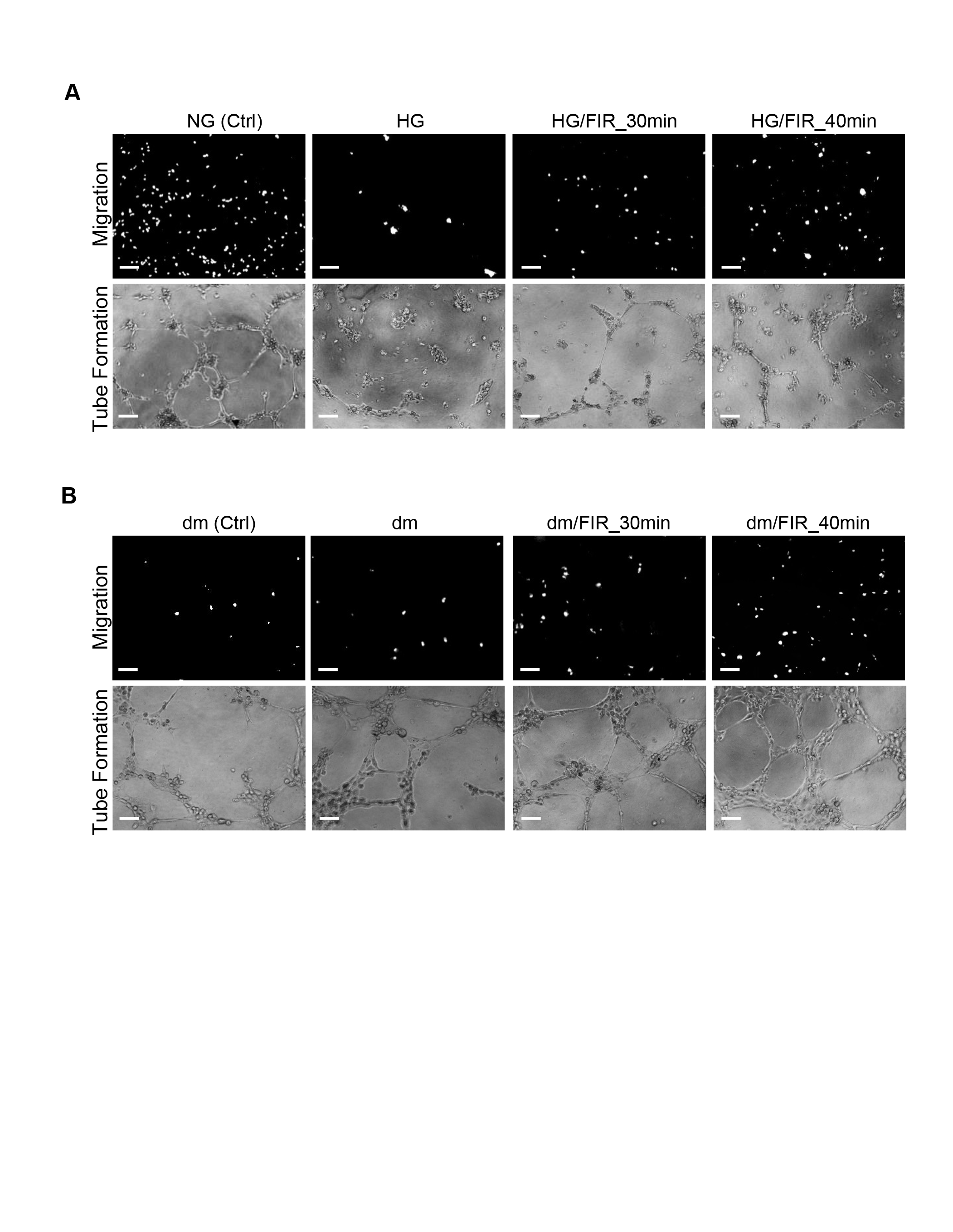

Supplement: S6 Fig — Scale bar: 50 μm. (TIF) [file pone.0147067.s006.tif]

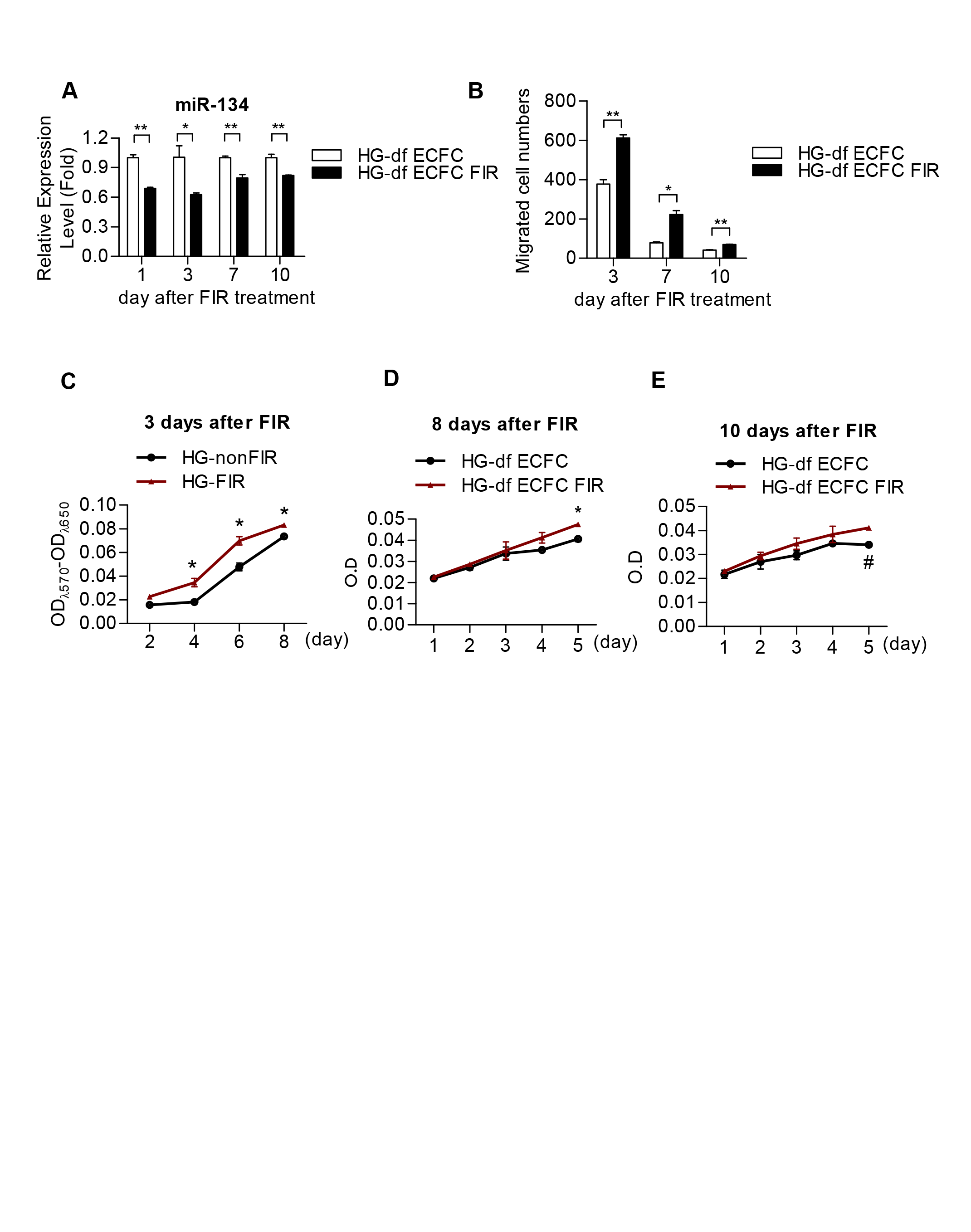

Supplement: S7 Fig — (A) The expression of miR-134 at indicated days after FIR treatment in HG-dfECFCs. * p < 0.05, ** p < 0.01 by one-way ANOVA followed by Tukey’s post-hoc test. (B) The quantative data of migrated cell numbers at indicated days after FIR treatment. * p < 0.05, ** p < 0.01 by one-way ANOVA followed by Tukey’s post-hoc test. (C~E) MTT cell proliferation assay performed after 3, 8 and 10 days after FIR treatment. * p < 0.05 by Mann-Whitney U test. (TIF) [file pone.0147067.s007.tif]

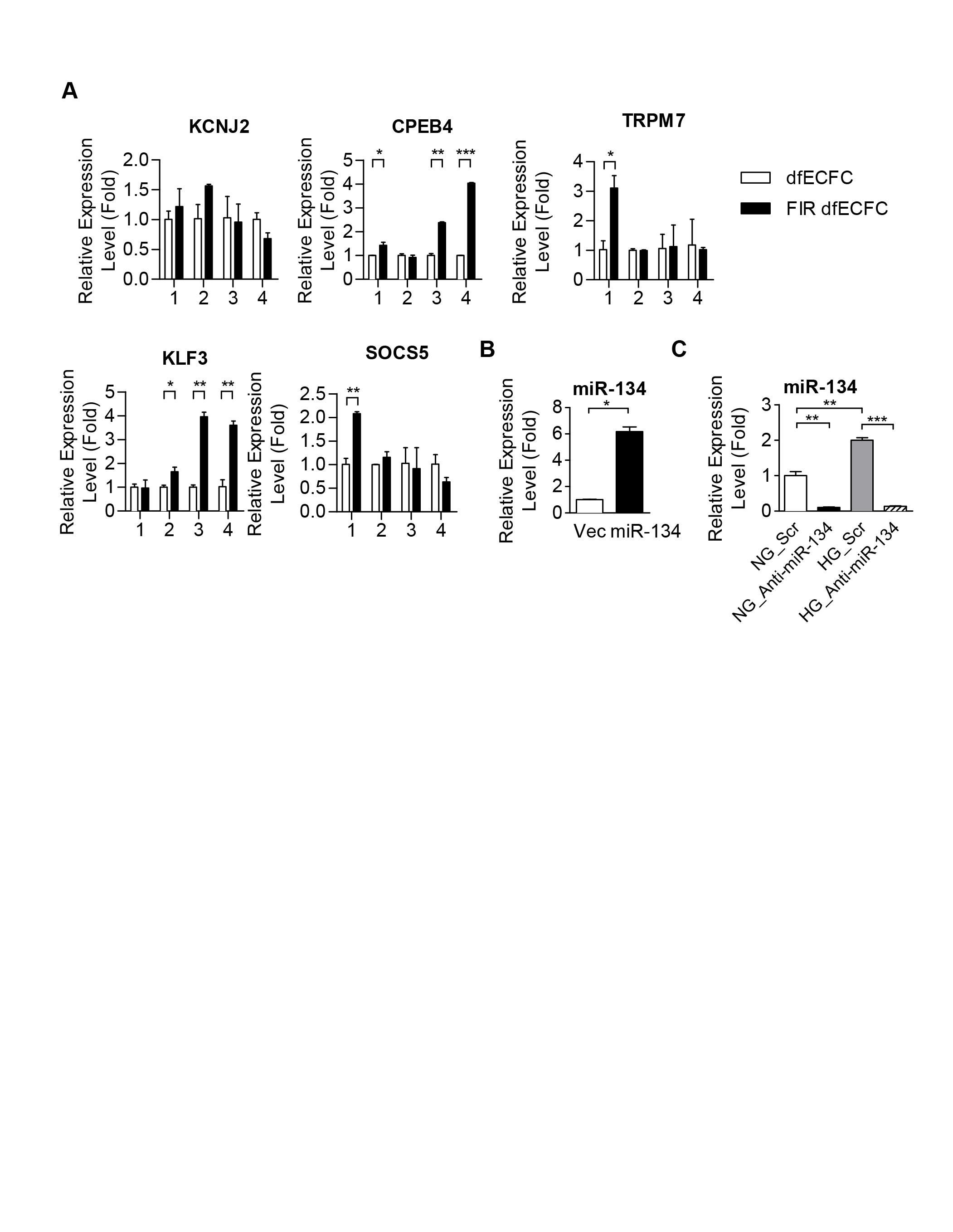

Supplement: S8 Fig — (A) Validation of the putative miR-134 target genes by RT-qPCR in dfECFCs with or without FIR treatment. * p < 0.05, ** p < 0.01, *** p < 0.001 by one-way ANOVA followed by Tukey’s post-hoc test. (B, C) The expression level of miR-134 in miR-134 overexpressed ECFCs (B) and miR-134 antagomir treated ECFCs (C). * p < 0.05 (B) by Mann-Whitney U test, ** p < 0.01, *** p < 0.001 (C) by one-way ANOVA followed by Tukey’s post-hoc test. (TIF) [file pone.0147067.s008.tif]

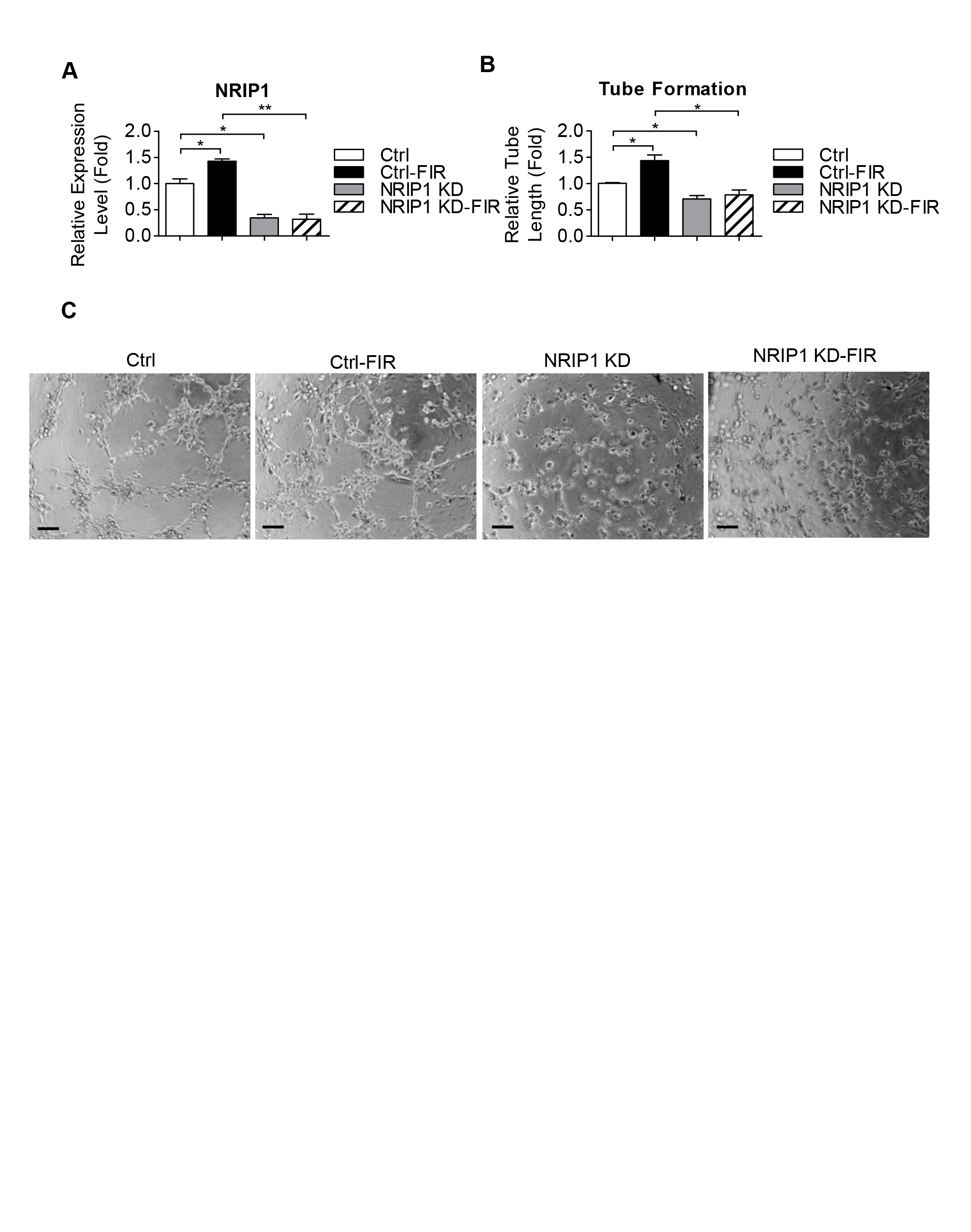

Supplement: S9 Fig — (A) The expression levels of NRIP1 in each group of ECFCs. * p < 0.05, ** p < 0.01 by one-way ANOVA followed by Tukey’s post-hoc test. (B) The quantitative data of tube formation assay. * p < 0.05 by one-way ANOVA followed by Tukey’s post-hoc test. (C) Representative images of the microvascular formation assays in each group cells. Scale bar: 50 μm. (TIF) [file pone.0147067.s009.tif]

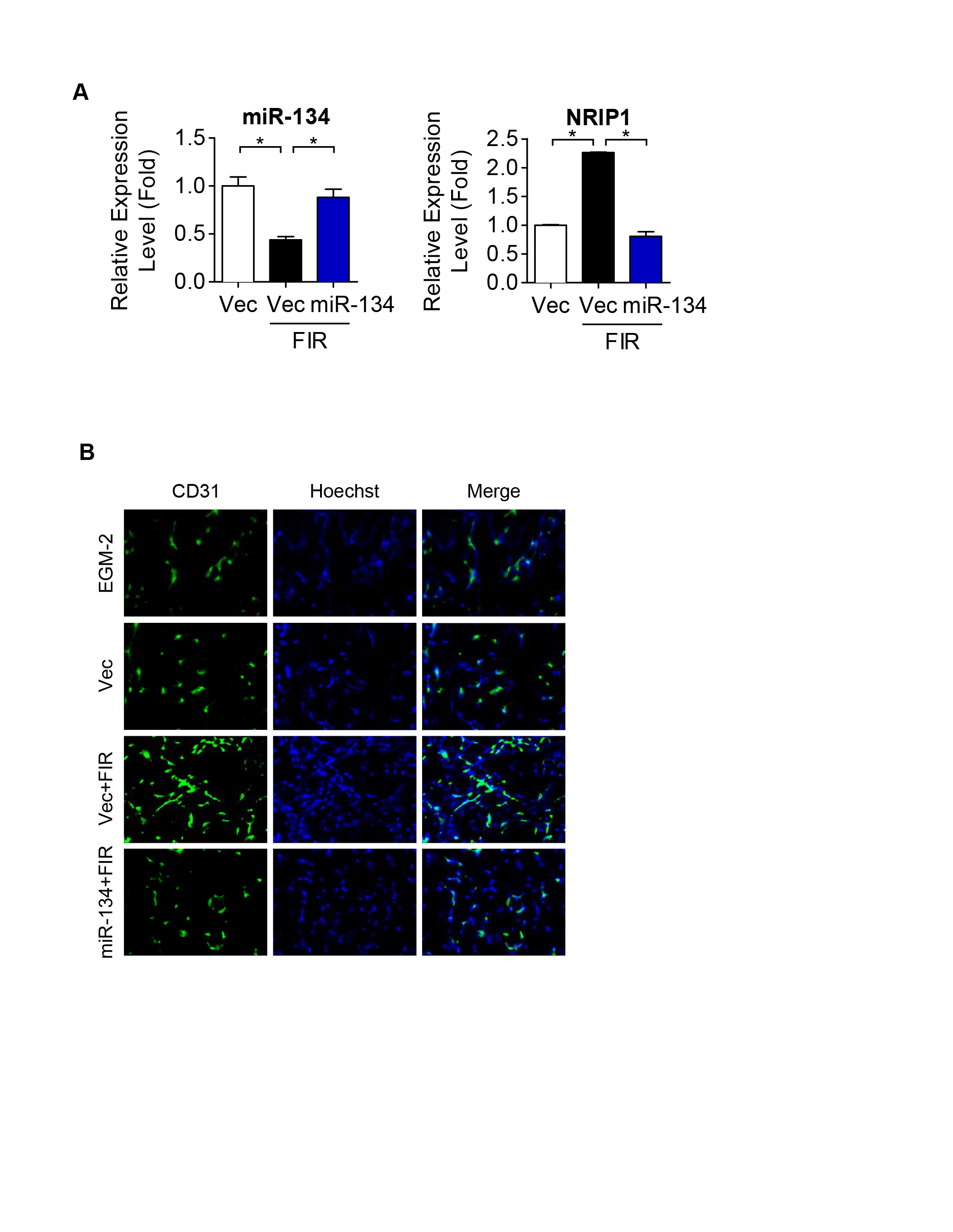

Supplement: S10 Fig — (A) Validation of the expression levels of miR-134 and NRIP1 in each group of ECFCs by RT-qPCR on day 0. * p < 0.05 by one-way ANOVA followed by Tukey’s post-hoc test. (B) Immunofluorescence staining of nude mice tissue samples on day 14 after injection with HG-dfECFCs. Capillaries in the ischemic muscles were visualized by anti-CD31 immunostaining (green), Hoechst: nuclear staining of live cells (blue). (TIF) [file pone.0147067.s010.tif]
